# A VEGFB-Based Peptidomimetic Inhibits VEGFR2-Mediated PI3K/Akt/mTOR and PLCγ/ERK Signaling and Elicits Apoptotic, Antiangiogenic, and Antitumor Activities

**DOI:** 10.3390/ph16060906

**Published:** 2023-06-20

**Authors:** Mohadeseh Namjoo, Hossein Ghafouri, Elham Assareh, Amir Reza Aref, Ebrahim Mostafavi, Ali Hamrahi Mohsen, Saeed Balalaie, Sylvain Broussy, S. Mohsen Asghari

**Affiliations:** 1 Department of Biology, University Campus II, University of Guilan, Rasht P.O. Box 14155-6619, Iran; 2 Department of Biology, Faculty of Sciences, University of Guilan, Rasht P.O. Box 14155-6619, Iran; h.ghafoori@guilan.ac.ir (H.G.); assareh.elham7@gmail.com (E.A.); 3 Belfer Center for Applied Cancer Science, Dana-Farber Cancer Institute, Harvard Medical School, Boston, MA 02115, USA; amir@xspherabio.com; 4 Department of Medicine, Stanford University School of Medicine, Stanford, CA 94305, USA; ebimsv@stanford.edu; 5 Institute of Biochemistry and Biophysics (IBB), University of Tehran, Tehran P.O. Box 1841, Iran; hamrahi.ali.1992@gmail.com; 6 Peptide Chemistry Research Center, K. N. Toosi University of Technology, Tehran P.O. Box 1841, Iran; balalaie@kntu.ac.ir; 7 CiTCoM, UMR CNRS 8038, U1268 INSERM, UFR de Pharmacie, Faculté de Santé, Université Paris Cité, 75006 Paris, France; sylvain.broussy@parisdescartes.fr

**Keywords:** peptidomimetic, VEGFR2, angiogenesis, tumor suppression, PI3K/Akt/mTOR signaling pathway, PLCγ/ERK signaling pathway

## Abstract

Vascular endothelial growth factor receptor 2 (VEGFR2) mediates VEGFA signaling mainly through the PI3K/AKT/mTOR and PLCγ/ERK1/2 pathways. Here we unveil a peptidomimetic (VGB3) based on the interaction between VEGFB and VEGFR1 that unexpectedly binds and neutralizes VEGFR2. Investigation of the cyclic and linear structures of VGB3 (named C-VGB3 and L-VGB3, respectively) using receptor binding and cell proliferation assays, molecular docking, and evaluation of antiangiogenic and antitumor activities in the 4T1 mouse mammary carcinoma tumor (MCT) model showed that loop formation is essential for peptide functionality. C-VGB3 inhibited proliferation and tubulogenesis of human umbilical vein endothelial cells (HUVECs), accounting for the abrogation of VEGFR2, p-VEGFR2 and, subsequently, PI3K/AKT/mTOR and PLCγ/ERK1/2 pathways. In 4T1 MCT cells, C-VGB3 inhibited cell proliferation, VEGFR2 expression and phosphorylation, the PI3K/AKT/mTOR pathway, FAK/Paxillin, and the epithelial-to-mesenchymal transition cascade. The apoptotic effects of C-VGB3 on HUVE and 4T1 MCT cells were inferred from annexin-PI and TUNEL staining and activation of P53, caspase-3, caspase-7, and PARP1, which mechanistically occurred through the intrinsic pathway mediated by Bcl2 family members, cytochrome c, Apaf-1 and caspase-9, and extrinsic pathway via death receptors and caspase-8. These data indicate that binding regions shared by VEGF family members may be important in developing novel pan-VEGFR inhibitors that are highly relevant in the pathogenesis of angiogenesis-related diseases.

## 1. Introduction

Vascular endothelial growth factors (VEGFs) are the primary regulators of endothelial cell (EC) functions, including angiogenesis and vascular permeability, in physiology and pathology [1,2]. The VEGF family is composed of VEGFA, VEGFB, VEGFC, VEGFD, VEGFE, VEGFF, and the placenta growth factor (PlGF). Although the development and regulation of normal blood vessels are influenced by different partners, VEGFA, often referred to as VEGF, is crucial for pathological angiogenesis.

The downstream signals of VEGFs are mediated by receptors, denoted VEGFR1, VEGFR2, and VEGFR3. VEGFA and VEGFB bind to VEGFR1; VEGFA binds to VEGFR2, and VEGFC and VEGFD bind to VEGFR3. In spite of high structural similarity, VEGFRs display different modes of activation, downstream signaling, and biological effects. Meanwhile, VEGFR2 mediates most of the VEGF-induced angiogenic signals. Notably, the function of the VEGF/VEGFR2 system is not limited to the EC function. It has become apparent that some tumor cells express VEGFR2 [3]. VEGF ligation to VEGFR2 promotes various intracellular signaling pathways, including PLCγ/ERK1/2, PI3K/AKT/mTOR, SRC, and small GTPases [4]. Moreover, VEGFR2 activates stress kinases and G protein-coupled receptors, which mediate the function of both endothelial and tumor cells [4].

Given the pivotal role of the VEGF/VEGFR2 signaling in tumor angiogenesis and development, suppression of VEGFR2-driven signaling pathways can inhibit angiogenesis and tumor metastasis. Several tyrosine kinase inhibitors, antibodies, and protein drugs are employed clinically to block VEGFR2-mediated signaling. Previous studies indicated that the blockade of both VEGFR1 and VEGFR2 could inhibit angiogenesis more effectively than the abrogation of VEGFR2 alone [5,6,7,8]. In recent years, peptides emerged as attractive drug discovery and development candidates. Their target specificity and potency, ease of synthesis, low toxicity, bioavailability, and the possibility of various chemical modifications render the peptides a new generation of therapeutics.

We have previously reported a peptide mimicking the loop2 region of VEGFB and its adjacent region in the 3D structure (named VGB3) that binds and suppresses VEGFR1 [9]. Here, we indicate that VGB3 can surprisingly bind and neutralizes VEGFR2 as well, and its antiangiogenic and antitumor properties depend on a disulfide loop formation. We also provide evidence that the cyclic structure of VGB3 potently suppresses PI3K/AKT/mTOR and PLCγ/ERK1/2 pathways in human umbilical vein endothelial cells (HUVECs) and 4T1 mammary carcinoma tumor (MCT) cells and induces apoptosis through both intrinsic and extrinsic pathways.

## 2. Results

### 2.1. Peptidomimetic Design

**Figure 1:**
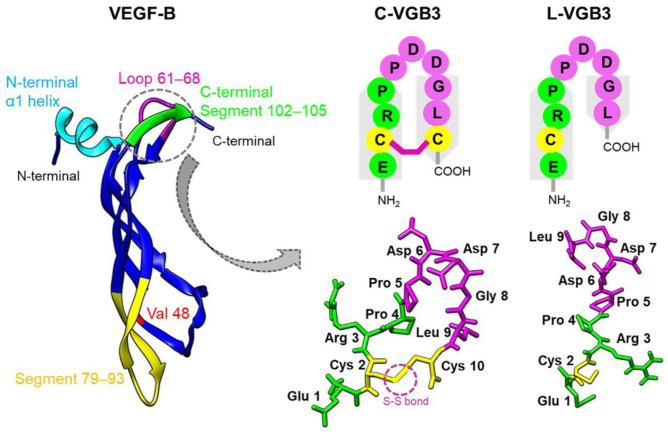
Graphical representation of VEGFB and two constructed VEGFB mimics. The ribbon presentation of VEGFB (PDB code: 2C7W) and five ligand binding segments identified in the VEGFB/VEGFR1 complex are represented on the left. The schematic representation and model structure of C-VGB3 and L-VGB3 constructed using MODELLER version 9.18 is shown on the right.

The regions of VEGFA involved in the binding to VEGFR2 are composed of helix α1 and loops 1–3. Similar epitopes are responsible for the binding of VEGFB to VEGFR1 in addition to the C-terminal segment of β6. Analysis of VEGFA binding to VEGFR2 using alanine scanning identified five side chains of VEGFA (Trp17, Gln46, Asp64, Gln79, and Ile83) accounting for most of the binding energy [10]. Notably, four out of five of these critical residues are either conserved in VEGFB (Gln79 and Ile83) or mutated to a highly similar residue (Phe17Trp and Glu64Asp). This high similarity proposes that peptidomimetics based on the interaction between VEGFB and VEGFR1 might also be the ligands of VEGFR2. Our recent study confirms this idea, where a cyclic peptidomimetic based on the helix α1 of VEGFB and a motif situated in its proximity bound to both VEGFR1 and VEGFR2 and inhibited angiogenesis and breast tumor growth [7]. Here, based on the same rationale, we designed a peptide based on the loop2 region of VEGFB and evaluated its VEGFR2 binding, functional properties, and mechanism of action. The loop2 region of VEGFB (^61^CPDDGLEC^68^) is very similar to that of VEGFA (^61^CNDEGLEC^68^). Spatially close to loop2, the C-terminal segment of β6, including residues ^102^ECRP^105,^ is implicated in the binding of VEGFB to VEGFR1 [11]. Accordingly, VEGFB segments ^62^PDDGLEC^68^ and ^102^ECRP^105^ were covalently linked into a single peptide as _2_HN-ECRPPDDGLC-COOH (Glu67 was removed) (Figures S1, S2 and S4). To mimic the U-shaped structure of the 61–68 region, a disulfide bond was incorporated between the cysteine residues (Figure 1, Figures S3 and S4). It has already been shown that this peptide, referred to as VGB3, can bind to VEGFR1 [9]. Here, we evaluated the binding of VGB3 to VEGFR2, the role of loop formation in its binding and antitumor properties, and its effect on the function and signaling pathways of the endothelial and tumor cell lines.

### 2.2. Loop Formation Is Essential for the Antitumor Activity of VGB3

**Figure 2:**
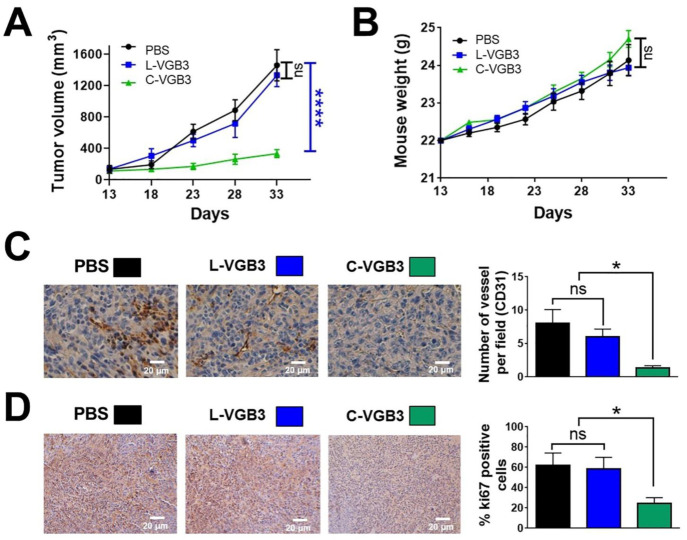
Loop formation is important for the antitumor activity of VGB3. (**A**) To investigate the efficacy of VGB3 peptides in vivo 13 days after transplantation of a 4T1 murine MCT into female BALB/c mice (the tumor volume ~150 mm^3^), the treatment groups received 0.2 mg·kg^−1^ (once every 2 days, i.v.) of either the C-VGB3 or L-VGB3 and the control group received PBS for 20 days. Error bars denote ± SEM. *n* = 6; **** *p* < 0.0001, ns = not significant; two-way ANOVA. (**B**) Investigation of C-VGB3 and L-VGB3 effects on mice weight. There were no significant differences between either C-VGB3 or L-VGB3 and control groups; two-way ANOVA. (**C**,**D**) Immunohistochemical (IHC) analyses of CD31 and Ki67 for the C-VGB3-, L-VGB3- and PBS-treated tumors. For IHC staining, the sections of tumor tissues were incubated with the primary mouse monoclonal antibodies for CD31 (1:2000 (*v*/*v*)) and Ki67 (5 µg·mL^−1^). CD31 (as an indicator of microvessel density) and Ki67 (a cellular marker of cell proliferation) significantly decreased in the C-VGB3-treated tumors compared with PBS-treated tumors. Photographs were taken at 40× magnification. Error bars denote ± SEM. *n* = 3; * 0.02 ≤ *p* ≤ 0.04, ns = not significant; one-way ANOVA.

As mentioned earlier, VGB3 contains a disulfide bond. To determine if loop structure is important for the activity of VGB3, two cyclic and linear peptide structures (C-VGB3 and L-VGB3, respectively) were synthesized (Figures S1–S5) and compared for antitumor activity using the murine 4T1 MCT model. Based on the previous study [9], peptides were administered once every two days intravenously (i.v.) at a dosage of 0.2 mg·kg^−1^. C-VGB3 inhibited 4T1 MCT by 76% (*p* < 0.0001), whereas L-VGB3 peptide had no effect (Figure 2A). Animals gained weight during three weeks of the peptides treatment (Figure 2B), suggesting that cyclic and linear peptides are not toxic at this dosage. We assessed tumor angiogenesis and cell proliferation to determine the antitumor mechanism of C-VGB3. Tumor angiogenesis was evaluated by staining of tumors (day 33 after implantation) for measurement of CD31-stained microvessels. Figure 2C shows a considerable decrease in vessel density after C-VGB3 treatment (84% reduction, *p* = 0.02), while L-VGB3 was similar to the phosphate-buffered saline (PBS)-treated control. The tumor cell proliferation was analyzed using Ki67 immunohistochemical staining as an index of tumor cell proliferation. C-VGB3 decreased Ki67-positive cells compared to the control (60%, *p* = 0.04), whereas L-VGB3 was ineffective (Figure 2D). These data suggest that VGB3 relies on loop structure for the antitumor and antiangiogenic activities.

### 2.3. Cyclic but Not Linear VGB3 Binds VEGFR2

**Figure 3:**
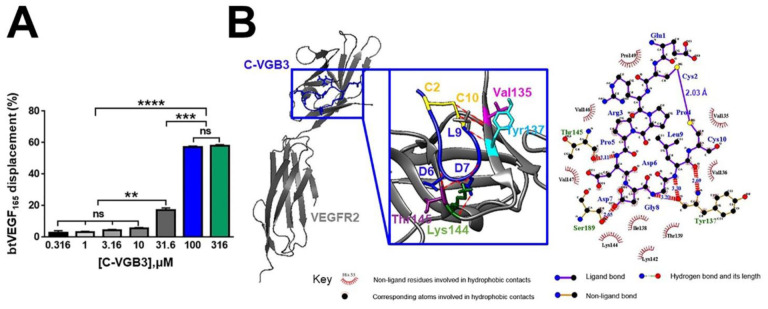
The binding affinity of C-VGB3 to VEGFR2. (**A**) ELISA-based displacement assay. The gradient concentrations of C-VGB3 (0, 0.316, 1, 3.16, 10, 31.6, 100, and 316 μM) were used in the assay. The results showed that C-VGB3 inhibits the binding of VEGF to the VEGFR2 extracellular domain in a dose-dependent manner. Error bars denote ± SEM. *n* = 3; ** *p* = 0.001, *** *p* = 0.0001, **** *p* < 0.0001, ns = not significant; one-way ANOVA. (**B**) A three-dimensional (produced by UCSF chimera software version 1.12) and a two-dimensional representation (produced by LigPlot software) of interactions in the C-VGB3/VEGFR2 complex is shown on the left and the right, respectively.

The significance of loop formation in VGB3 activity was further elucidated by the examination of the binding of linear and cyclic structures to the extracellular domain of VEGFR2. Competition binding assay showed that C-VGB3 inhibits the binding of biotinylated VEGF (bt-VEGF) to KDR dose-dependently (Figure 3A). In contrast, L-VGB3 exhibited no measurable binding even at a high concentration (3 mM) (Figure S6).

The capabilities of cyclic and linear peptides to bind the extracellular domain of VEGFR2 were also compared by docking analyses using PatchDock and HADDOCK web servers. Results indicated that the disulfide bond contributes to a strong interaction of the peptide with the receptor. A comparison of the atomic contact energies of the top solutions obtained by PatchDock suggested a much more stable complex with VEGFR2 for C-VGB3 than for L-VGB3 (Table 1). Likewise, analyses using the HADDOCK server suggested a noticeably increased total interaction in the C-VGB3/VEGFR2 complex (Figure 3B) compared to L-VGB3/VEGFR2 complex (Table 1 and Table S1). According to HADDOCK results, the electrostatic interactions play an important role in the binding of C-VGB3 to VEGFR2 (Table 1), as this is predicted by the presence of residues with polar side chains in the C-VGB3, including Glu1, Arg3, Asp6, and Asp7. In addition, the hydrophobic interactions play a considerable role in C-VGB3 binding to VEGFR2. As shown in Figure 3B and Table S1, 80% of C-VGB3 residues participate in the hydrophobic interactions. Moreover, several H-bonds were predicted between C-VGB3 and VEGFR2 (Figure 3B and Table S1). The electrostatic (−33.2 *±* 14.6 kcal·mol^−1^) and hydrophobic (−32.3 *±* 1.2 kcal·mol^−1^) interactions have an approximately equal contribution to the production of the L-VGB3/VEGFR2 complex (Table 1). The role of electrostatic interactions in the C-VGB3/VEGFR2 complex (−143.5 *±* 21.7 kcal·mol^−1^) is considerably more than in the L-VGB3/VEGFR2 complex (−33.2 *±* 14.6 kcal·mol^−1^). In addition, the C-VGB3/VEGFR2 complex exhibited a higher docking score and total interaction energy (−67.7 ± 4.9 and −184 ± 26.9 kcal·mol^−1^, respectively) compared to those of the L-VGB3/VEGFR2 complex (−47.8 ± 4.7 and −65.5 ± 15.8 kcal·mol^−1^, respectively). According to the docking analyses, L-VGB3 cannot form a stable complex with VEGFR2, but the cyclic structure results in a strong affinity toward the ectodomain of the receptor.

### 2.4. C-VGB3 Inhibited EC Proliferation and Tube Formation through Abrogation of VEGFR2-Mediated Signaling Pathways

**Figure 4:**
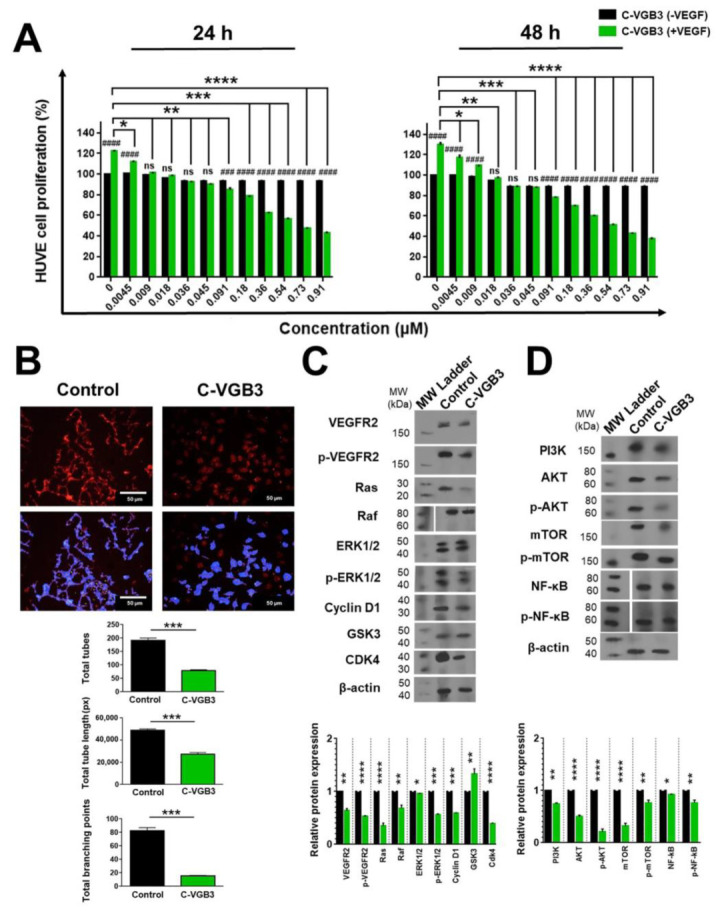
Inhibition of EC proliferation and tubulogenesis by C-VGB3 through abrogation of VEGFR2-mediated signaling pathways. (**A**) MTT assay was performed to investigate C-VGB3 effect on the proliferation of ECs. C-VGB3 (0–0.91 μM) was used with (20 ng·mL^−1^) or without VEGF. The control cells were treated with no peptide. After 24 and 48 h, the absorbance was measured at 570 nm utilizing an ELISA plate reader (Space Fax 2100, Awareness, Westport, CT, USA). Error bars indicate ± SEM. *n* = 3; * *p* = 0.02, ** *p* = 0.001, *** and ### *p* = 0.0001, **** and #### *p* < 0.0001, ns = not significant; one-way ANOVA. (**B**) The anti-tubulogenesis effect of C-VGB3 in VEGF (20 ng·mL^−1^)-induced HUVECs. Image analysis performed by Wimasis image analysis software is illustrated in the bottom panel. Error bars indicate ±SEM. *n* = 3; *** 0.0004 ≤ *p* ≤ 0.0001; unpaired Student’s *t*-test. To evaluate the mechanism by which C-VGB3 diminished cell proliferation and tube formation, Western blot analysis was performed for HUVECs. (**C**) The results showed that C-VGB3 could significantly decline VEGFR2 expression and phosphorylation, thereby significantly decreasing the VEGF (20 ng·mL^−1^)-induced expression or phosphorylation of signaling proteins RAS, RAF, ERK 1/2, Cyclin D1, and Cdk4. The peptide also increased the expression of GSK3, an antiproliferative protein. Error bars indicate ± SEM. *n* = 3; * *p* = 0.02, ** *p* = 0.001, *** *p* = 0.0001, **** *p* < 0.0001; one-way ANOVA. (**D**) The results showed that C-VGB3 could significantly decrease PI3K, AKT, p-AKT, mTOR, p-mTOR, and NF-κB. Error bars indicate ± SEM. *n* = 3; * *p* = 0.02, ** *p* = 0.001, **** *p* < 0.0001; one-way ANOVA.

Since the blockade of VEGFR2 inhibits different aspects of angiogenesis, we investigated the peptide effect on EC proliferation and tube formation after VEGF (20 ng·mL^−1^) stimulation. Treatment of HUVECs with increasing concentrations of C-VGB3 (0–0.91 μM) for 24 and 48 h dose-dependently decreased the cell viability (Figure 4A) with a half-inhibitory concentration (IC_50_) of 0.51 and 0.38 μM, respectively. Next, the antiangiogenic activity of C-VGB3 was evaluated using a tube formation assay. As shown in Figure 4B, C-VGB3 (0.40 μM) could inhibit VEGF-induced tubulogenesis of ECs as a statistically significant decreased tube numbers, tube length, and tube branching points by 58.64, 44.37, and 81.46%, respectively, compared to the control (*p* < 0.001). These data indicate that C-VGB3 potently inhibits VEGF-induced tubulogenesis of ECs. 

Next, to explore the mechanisms of the antiangiogenic property of C-VGB3, canonical signaling pathways mediated by VEGFR2 were assessed in HUVECs after 24 h incubation with C-VGB3 (0.40 μM). The binding of VEGF to VEGFR2 causes autophosphorylation of its tyrosine kinase domain and increases the receptor′s expression level on the cell surface [12,13]. Therefore, we evaluated the ability of C-VGB3 to suppress the levels of constitutive and phosphorylated VEGFR2 in HUVECs. As indicated in Figure 4C, treatment of HUVECs with C-VGB3 markedly inhibited the constitutive level of VEGFR2 (*p* = 0.001) as well as the formation of p-VEGFR2 (*p* < 0.0001) compared to untreated (0 μM C-VGB3) controls.

Since canonical activation of VEGFR2 turns on PLCγ/ERK1/2 and PI3K/AKT/mTOR signaling pathways, we examined the effects of C-VGB3 treatment on these pathways. The Ras/RAF/MEK/ERK1/2 signaling cascade regulates cellular proliferation, migration, differentiation, and survival [4,14,15]. Subsequently, CyclinD1 and cyclin-dependent kinase (CDK)-2/-4 are transcribed and activated through ERK, leading to the progression of the cell cycle through the G1 phase [16]. Hence, the molecular mechanism by which C-VGB3 inhibits cell proliferation was investigated by measuring the expression level of RAS, RAF, CyclinD1, GSK3, CDK4, ERK1/2, and p-ERK1/2. As indicated in Figure 4C, C-VGB3 decreased RAS (*p* < 0.0001), RAF (*p* = 0.001), ERK1/2 (*p* = 0.02), p-ERK1/2 (*p* = 0.0001), CyclinD1 (*p* = 0.0001), and CDK4 (*p* < 0.0001) compared to untreated controls, indicative of the downregulation of PLCγ/ERK1/2 pathway. Additionally, the antiproliferative effect of C-VGB3 was confirmed by an increased expression of GSK3, as an antiproliferative protein, compared to the control (*p* = 0.001) (Figure 4C).

VEGFR2-driven PI3K/AKT/mTOR signaling pathway is involved in various EC functions, including proliferation, adhesion, survival, migration, invasion, metabolism, and angiogenesis [17]. Accordingly, we analyzed PI3K, AKT, p-AKT, mTOR, and p-mTOR in HUVECs after C-VEGB3 treatment. The PI3K expression level decreased after treatment with C-VGB3 compared to the control (*p* = 0.001) (Figure 4D). Consequently, AKT and p-AKT levels in EC were markedly reduced (*p* < 0.0001) (Figure 4D). AKT regulates physiological and pathological angiogenesis, and vascular metabolism via the mTOR complex [17]. We observed that C-VGB3 treatment strongly suppressed the total expression of mTOR (*p* < 0.0001) and the phosphorylation of mTOR (*p* = 0.001) compared with the controls (Figure 4D). NF-κB has been implicated in various aspects of angiogenic signaling by regulating hypoxia, survival, oxidative stress, and the production of pro-angiogenic and pro-inflammatory cytokines, and MMPs [18,19]. Analysis of HUVECs indicated that the expression of NF-κB decreased after treatment of C-VGB3 compared to the controls (*p* = 0.02). In addition, C-VGB3 resulted in suppressed NF-κB phosphorylation compared with the untreated ECs (*p* = 0.001) (Figure 4D).

### 2.5. C-VGB3 Inhibited Proliferation and VEGFR2 Signaling Pathways in 4T1 MCT Cells

**Figure 5:**
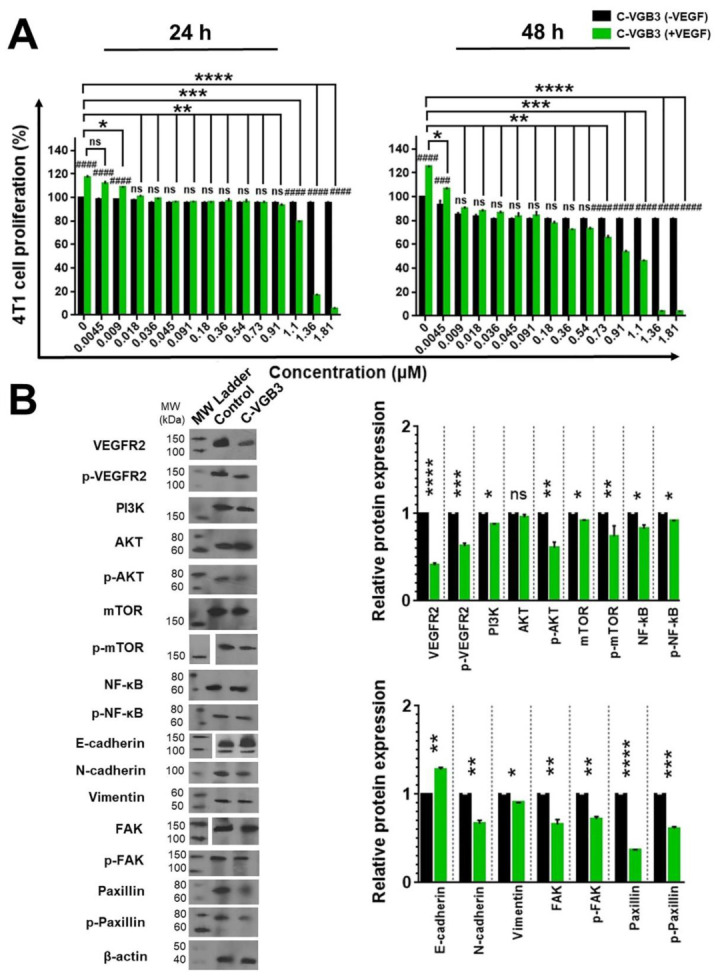
Suppression of 4T1 cell proliferation and metastasis by C-VGB3 through blockade of VEGFR2 signaling pathways. (**A**) MTT assay was performed to investigate C-VGB3 effect on the proliferation of 4T1 tumor cells in vitro. C-VGB3 was used at concentrations 0–1.81 μM in the presence (20 ng·mL^−1^) or the absence of VEGF. The control cells were treated with no peptide. After 24 and 48 h, the absorbance was measured at 570 nm utilizing an ELISA plate reader (Space Fax 2100, Awareness, Westport, CT, USA). Error bars represent ± SEM. *n* = 3; * *p* = 0.0 2, ** *p* = 0.001, *** and ### *p* = 0.0001, **** and #### *p* < 0.0001, ns = not significant; one-way ANOVA. (**B**) To evaluate the mechanism by which C-VGB3 diminished cell proliferation and metastasis, Western blot analysis was performed for 4T1 cells. The results showed that C-VGB3 could significantly decline VEGFR2 expression and phosphorylation, thereby significantly decreasing the VEGF (20 ng·mL^−1^) induced expression or phosphorylation of signaling proteins implicated in cell proliferation and metastasis, including PI3K, p-Akt, p-mTOR, mTOR, NF-κB, Vimentin, N-cadherin, FAK, and paxillin. Furthermore, treatment with the peptide significantly increased E-cadherin protein expression. Error bars represent ± SEM. *n* = 3; * *p* = 0.02, ** *p* = 0.001, *** *p* = 0.0001, **** *p* < 0.0001, ns = not significant; one-way ANOVA.

We have previously indicated that the 4T1 MCT cell expresses VEGFR2 and is an appropriate model for the investigation of VEGFR2-blocking peptides [5,6,7,8,11]. It raises the possibility that the antitumor property of C-VGB3 (Figure 2) is also related to a direct effect on tumor cells. Therefore, we examined the effect of peptides on this cell line. After 24 and 48 h treatment, C-VGB3 inhibited the proliferation of 4T1 MCT cells with IC_50_ values of 0.56 and 0.37 μM, respectively (Figure 5A). The antiproliferative activity of C-VGB3 on 4T1 cells suggests that the tumor growth regression (Figure 2A) is a combination effect arising from the antiangiogenic property as well as direct inhibition of tumor cells.

Next, VEGFR2 signaling was investigated in 4T1 MCTs. Western blotting showed that treatment of 4T1 cells with C-VGB3 (0.40 μM) suppressed VEGFR2 (*p* < 0.0001) and p-VEGFR2 (*p* = 0.0001) compared with untreated cells (Figure 5B). Considering the implication of the PI3K/AKT/mTOR signaling pathway in most human cancers [17], the effect of C-VGB3 on this pathway was evaluated in 4T1 MCT cells. As shown in Figure 5B, C-VGB3 (0.40 μM) significantly decreased the levels of PI3K (*p* = 0.02), p-AKT (*p* = 0.001), mTOR (*p* = 0.02), p-mTOR (*p* = 0.001), compared to untreated controls. The NF-κB pathway is an important target of AKT, which contributes to abnormal cell proliferation and differentiation, the inhibition of apoptosis, enhanced metastasis, and treatment resistance [20]. Therefore, the inhibition of upstream AKT signaling would be expected to downregulate NF-κB and/or p-NF-κB. Total protein extract lysates from C-VGB3-treated and untreated 4T1 cells showed decreased levels of NF-κB and p-NF-κB in the peptide-treated group compared with untreated controls (*p* = 0.02) (Figure 5B).

VEGFR2 signaling is involved in the promotion of metastasis in a variety of tumor cells [21,22]. Therefore, we examined the effects of C-VGB3 on the levels of metastatic factors, including N-cadherin, E-cadherin, vimentin, FAK, p-FAK, paxillin, and p-paxillin. Upregulated NF-κB induces metastatic-related factors [23] and the epithelial–mesenchymal transition (EMT) process. EMT is associated with the progression to more aggressive cancer phenotypes through increased tumor cell mobility, invasiveness, and resistance to apoptotic stimuli [24]. The hallmark of EMT is the upregulation of vimentin and N-cadherin and concomitantly the downregulation of E-cadherin. Compared to controls, C-VGB3 potently increased the E-cadherin expression (*p* = 0.001) and reduced the level of N-cadherin (*p* = 0.001) and vimentin (*p* = 0.02). The FAK/paxillin signaling cascade is implicated in cell adhesion, migration, proliferation, and survival. Therefore, the downregulation of this axis is a marker of metastasis inhibition [25]. Analysis of 4T1 cell lysates indicated that C-VGB3 substantially inhibited FAK (*p* = 0.001), p-FAK (*p* = 0.001), paxillin (*p* < 0.0001), and p-paxillin (*p* = 0.0001) compared to controls.

### 2.6. C-VGB3 Induces Endothelial and Tumor Cell Apoptosis

**Figure 6:**
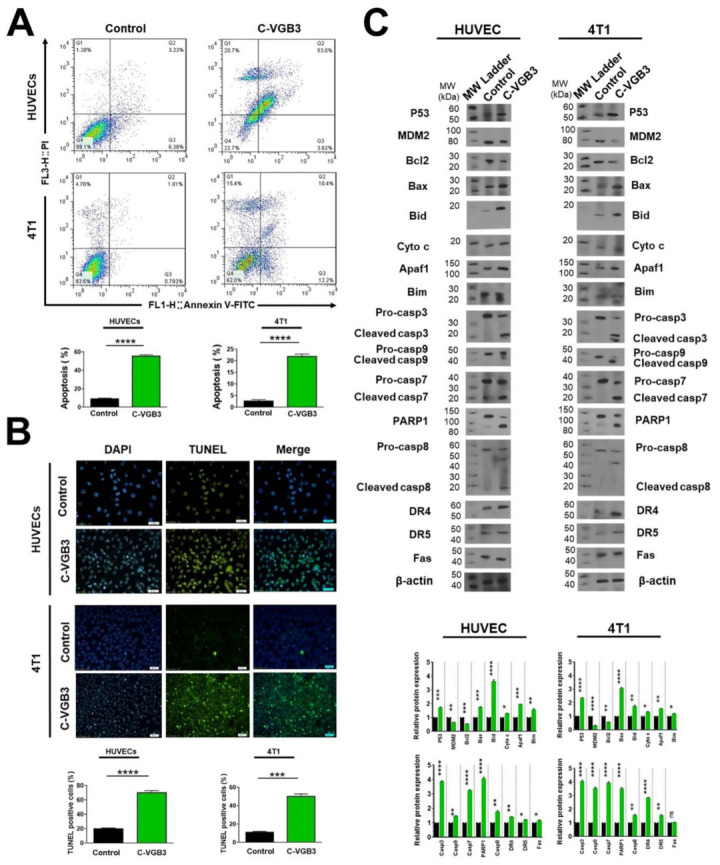
Inducing apoptosis in endothelial and tumor cells treated with VEGF by C-VGB3 peptide in vitro. Three different procedures were performed to investigate the effects of C-VGB3 on apoptosis induction. (**A**) Investigating the effects of C-VGB3 on HUVE and 4T1 cell survival using staining with annexin V and PI, followed by flow cytometry analysis. Quantitative analysis of apoptosis is shown in the bottom panel. C-VGB3 (0.40 μM) significantly inhibited anti-apoptotic effects induced by VEGF (20 ng·mL^−1^). Error bars signify ± SEM. *n* = 3; **** *p* < 0.0001; unpaired Student’s *t*-test. (**B**) The TUNEL assay was performed on the HUVE and 4T1 cells using TUNEL Assay Kit. Analyses acquired by ImageJ are illustrated in the bottom panel. Error bars denote ± SEM. *n* = 3; *** *p* = 0.0002, **** *p* < 0.0001; unpaired Student’s *t*-test. (**C**) To evaluate the pro-apoptotic effects of C-VGB3, Western blot analysis was performed for HUVE and 4T1 cells. The results showed that C-VGB3 could significantly increase the expression and/or phosphorylation of pro-apoptotic proteins and decrease the VEGF (20 ng·mL^−1^) induced expression or phosphorylation of anti-apoptotic proteins in both cell lines. Error bars denote ± SEM. *n* = 3; * *p* = 0.02, ** *p* = 0.001, *** *p* = 0.0001, **** *p* < 0.0001, ns = not significant; One-way ANOVA.

An important consequence of VEGF signals is cell survival dominantly via PI3K/AKT/mTOR pathway. Thus, inhibition of VEGF signals leads to the induction of apoptosis. Accordingly, the ability of C-VGB3 to induce apoptosis was investigated. First, apoptotic endothelial and tumor cells were analyzed by flow cytometry after staining with annexin V-FITC and PI. (Figure 6A). Treatment with C-VGB3 (0.40 μM) induced significant apoptosis in both cell lines compared to the controls. The level of apoptotic cells was significantly higher than in the control group (HUVECs: 56.62 vs. 9.61, *p* < 0.0001; and 4T1: 22.60 vs. 1.80, *p* < 0.0001) after 24 h of treatment. Apoptosis induction by C-VGB3 was further characterized by the TUNEL assay, as an indicator of late apoptosis, with a confocal microscope (Figure 6B). The results showed that C-VGB3-treated cells are fluorescent-labeled more than control cells (HUVECs, *p* < 0.0001; and 4T1 cells, *p* = 0.0002).

To elucidate the apoptotic mechanism of C-VGB3, we assessed the intrinsic and extrinsic signaling pathways of apoptosis. P53 is known as a pro-apoptotic protein. AKT negatively controls P53 levels by inducing MDM2-mediated targeting of P53 for degradation [26]. Therefore, the downregulation of AKT dissociates the P53 and MDM2 complex. We observed that P53 levels were enormously increased in C-VGB3-treated ECs (*p* = 0.0001) and tumor cells (*p* < 0.0001) compared with controls. Concurrently, MDM2 levels were markedly decreased in HUVECs (*p* = 0.001) and 4T1 cells (*p* < 0.0001). Many studies have shown that VEGF-mediated activation of the PI3K/AKT/mTOR pathway contributes to cell survival through the Bcl2 family of proteins such as Bcl2, Bax, and Bid. On the other hand, P53 directly interacts with members of the Bcl2 family and influences apoptosis [27,28]. Bax is a pro-apoptotic member in the Bcl2 family that mediates mitochondrial permeabilization and, therefore, the intrinsic cell death pathway. It has been shown that Bcl2 binds to Bax and neutralizes its apoptotic effect. Thus, the Bax/Bcl2 ratio is crucial for determining whether cells will undergo apoptosis or survive. As indicated in Figure 6C, C-VGB3 induced a decrease in Bcl2 expression in HUVE and 4T1 cells (*p* = 0.0001 and *p* = 0.001, respectively), and concomitantly increased Bax level (*p* = 0.0001 and *p* < 0.0001, respectively). These effects resulted in an approximately 3.35- and 5.25-fold increase in Bax/Bcl2 ratio in HUVE and 4T1 cells, respectively, which favors apoptosis via cytochrome c followed by caspase activation through oligomerization of Apaf1. In HUVECs, C-VGB3 treatment led to an increase in cytochrome c (*p* = 0.02) and Apaf1 (*p* = 0.0001) compared to controls (Figure 6C). Similarly, C-VGB3 promoted cytochrome c release (*p* = 0.02) and Apaf-1 (*p* = 0.001) in 4T1 cells (Figure 6C). Bid is a pro-apoptotic protein that migrates to mitochondria and acts as a sentinel for protease-mediated death signals [29]. Bid expression level increased after C-VGB3 treatment in HUVECs (*p* < 0.0001) and 4T1 cells compared to controls (*p* = 0.001) (Figure 6C). Bim is known as a pro-apoptotic factor that is attenuated in response to VEGFR2 signaling [30]. As indicated in Figure 6C, Bim levels increased in both ECs and tumor cells after C-VGB3 treatment (*p* = 0.001 and *p* = 0.02, respectively).

To evaluate the participation of different caspases in the apoptotic effect of C-VGB3, we assessed the levels of pro-caspases and cleaved caspases in C-VGB3-treated HUVE and 4T1 cells. Results of Figure 6C show increased levels of cleaved caspase-3 (3.8- and 4.0-times), cleaved caspase-9 (1.4- and 3.5-times), and cleaved caspase-7 (3.3- and 4.1-times) in C-VGB3-treated HUVE and 4T1 cells, respectively. Hence, C-VGB3 could induce apoptosis in the endothelial and tumor cells via the induction of caspases. Altogether, the involvement of P53, Bcl2 family members (including Bcl2, Bax, Bid), the release of cytochrome c, activation of Apaf1 and caspases-9, caspase-3, and caspase-7 suggest that the mitochondrial pathway is engaged in the induction of apoptosis.

During apoptosis induction, DNA fragmentation results in PARP1 activation; therefore, its cleavage is an important biomarker of apoptosis. Western blot analysis indicated a 3.50- and 4.05-fold increase in the level of cleaved PARP1 in C-VGB3-treated cells compared to controls, respectively (*p* < 0.0001) (Figure 6C).

Importantly, we also observed that C-VGB3 led to the activation of caspase-8. In both cell lines, lower amounts of the cleaved caspase-8 were detected after treatment with C-VGB3 compared to controls (*p* = 0.001) (Figure 6C). Given that caspase-8 is involved in the death receptor-mediated extrinsic apoptotic signaling pathway, we sought to determine whether C-VGB3 sensitizes cells in a receptor-specific manner. The cell surface death receptor 4 (DR4 or CD261) and death receptor 5 (DR5 or CD262) induce apoptosis after ligation to TRAIL. Another death receptor is Fas (CD95), which can mediate apoptosis induction when bound to its natural ligand. We investigated whether DR4, DR5, and Fas are involved in the C-VGB3-driven apoptosis in HUVE and 4T1 cell lines. In both endothelial and tumor cell lines, C-VGB3 led to an increase in the level of DR4 (*p* = 0.001 and *p* < 0.0001) and DR5 (*p* = 0.02 and *p* = 0.001) compared to the untreated cells. Fas expression, however, was slightly increased in HUVECs (*p* = 0.02) but not affected in 4T1 cells after C-VGB3 treatment. These results suggest that, as a result of C-VGB3 treatment, death receptors, especially DR4, and DR5 recruited caspase-8 to form the death-inducing signaling complex.

**Figure 7:**
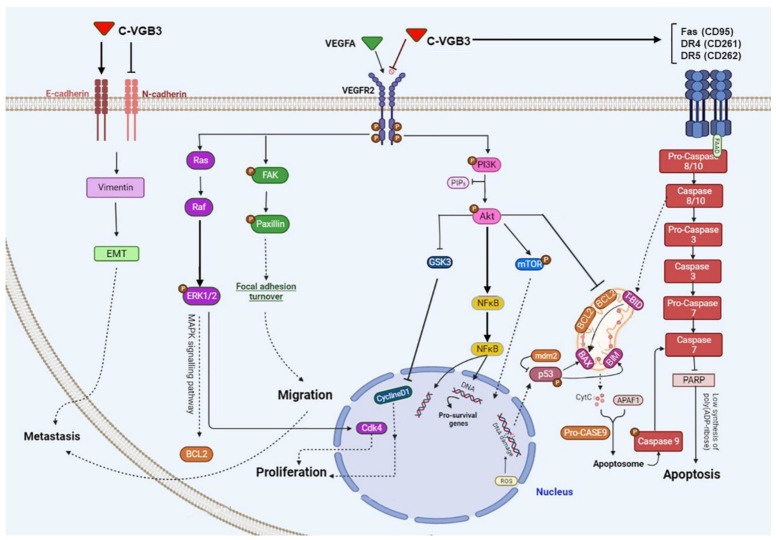
Schematic representation of the effect of cyclic C-VGB3 on VEGFR2 downstream signaling pathways. C-VGB3 recognizes the extracellular domain of VEGFR2 and inhibits its activation (phosphorylation) and downstream signaling pathways in endothelial and/or MCT cells.

Figure 7 shows the wide range of VEGFR2 downstream signaling mediators targeted by C-VGB3 in HUVEC and/or 4T1 MCT cells. As indicated in this figure, three metabolic alterations caused by VEGFR2 suppression: abrogation of PI3K/AKT/mTOR pathway, leading to angiogenesis inhibition and apoptosis promotion in endothelial and tumor cells; suppression of PLCγ/ERK1/2 pathway, resulting in antiproliferative effects; and suppression of metastasis-related mediators in 4T1 MCT cells.

## 3. Discussion

VEGF/VEGFR2 system is detected in diverse cancers and correlates to tumor development and metastasis. The activation of VEGFR2 turns on canonical PI3K/AKT/mTOR and PLCγ/ERK1/2 signaling pathways [17,31]. Importantly, downstream signaling of VEGFR2 has been implicated not only in tumor ECs but also in the other players of the tumor microenvironment. Therefore, it has been an attractive target for developing antiangiogenic and anticancer drugs. Peptides bind to target proteins with high specificity. In addition, tumor-targeting peptides are recognized for their unique tumor-penetrating properties, suggesting that they have the potential to be inhibitors of extracellular proteins such as VEGFRs in tumors.

Our previous study has shown that a peptide reproducing helix α1 of VEGFB can unexpectedly interfere with VEGF binding to VEGFR2 and, consequently, inhibit VEGF-stimulated angiogenesis and tumor growth [7]. In the present study, we extend this observation using another peptide (C-VGB3) that mimics loop2 and its adjacent region of VEGFB. C-VGB3 has been shown to bind and neutralize VEGFR1 [9]. According to the results of the current study, C-VGB3 also shows a strong binding affinity toward the extracellular domain of VEGFR2. For instance, competitive binding of C-VGB3 (100 μM) with biotinylated VEGFA to the ectodomain of VEGFR1 [9] and VEGFR2 (current study) resulted in 63 and 58% displacement of btVEGF165, respectively. It is interesting to note that a peptidomimetic based on VEGFB not only recognizes VEGFR1 but also binds to VEGFR2. This dual specificity is likely due to high sequence similarity between VEGFA and VEGFB in the loop2 region (^61^CNDEGLEC^68^ in VEGFA vs. ^61^CPDDGLEC^68^ in VEGFB). Comparative analyses of L- and C-VGB3 indicated that its receptor binding, antiproliferative, antiangiogenic, and antitumor properties rely on the formation of a loop structure because these properties are abolished in the linear peptide. Considering that the loop2 region in VEGFB adopts a U-shaped structure [32], these data suggest that the antagonistic property of C-VGB3 is due to preserving the loop structure. Several investigations have also shown that the loop structure is important for the activity of antitumor peptides [33,34,35].

Detailed cell signaling studies demonstrate that C-VGB3 potently inhibited the expression and activation (phosphorylation) of VEGFR2 on the surface of ECs, thereby downregulating PLCγ/ERK1/2 and PI3K/AKT/mTOR signaling pathways. Thus, inhibition of EC proliferation and tubulogenesis in vitro and angiogenesis in vivo is attributed to the suppression of these canonical downstream signaling pathways. These results follow previous investigations, which indicate that suppression of VEGFR2 significantly inhibits angiogenesis through downregulation of the PLCγ-ERK1/2 pathway [36,37,38,39,40] and PI3K/AKT/mTOR pathway [5,6,7,17,36,41]. Notably, targeting VEGFR1, which acts primarily via the PI3K/AKT signaling pathway, can improve angiogenesis therapy. Thus, owing to cross-activation and pathway convergence, co-targeting of VEGFR1 and VEGFR2 inhibits angiogenesis more effectively than blocking VEGFR2 alone [5,6,7,8,11,42,43]. Because C-VGB3 can bind and suppress VEGFR1 [9], the inhibition of EC function by C-VGB3 could be enhanced by its effect on VEGFR1. In addition to ECs, VEGF/VEGFR2 signaling occurs in tumor cells and the other stromal cells, which contributes to tumorigenesis [3]. VEGFR1 mainly acts through PI3K/AKT/mTOR [44]. Thus, the observed effects of C-VGB3 on the PLCγ-ERK1/2 signaling pathway are related to its VEGFR2-neutralizing property. An important aspect of our study is related to the direct effect of C-VGB3 on tumor cell signaling through the VEGF/VEGFR2 system. Several studies showed that a correlation exists between the expression of VEGF/VEGFR2 and metastasis in different cancers [3,45]. We showed that VEGFR2-mediated signaling contributes to metastasis-related factors and cell survival in 4T1 MCT cells. Previous studies indicated that VEGF could stimulate EMT in carcinoma cells [46,47]. Results of the current study confirm that VEGF promotes EMT in VEGFR2-expressing tumor cells, and suppression of the VEGF/VEGFR2 system may attenuate this process in favor of metastasis inhibition. On the other hand, FAK/Paxillin signaling axis is involved in VEGF-driven metastasis-related responses [25]. We indicated that C-VGB3 could efficiently suppress cell detachment by the marked inhibition of the FAK/paxillin signaling axis, which is an initial step in the metastatic transformation. We also provide evidence that the blockade of pro-survival signals of the VEGF/VEGFR2 system by C-VGB3 results in apoptosis induction through both intrinsic and extrinsic pathways. Induction of apoptosis as a function of disturbed VEGFR2 signaling has been reported in ECs [48,49] and tumor cells [8,41,50].

## 4. Materials and Methods

### 4.1. Cell Culture

Tumor and endothelial cell lines (the National Cell Bank, Pasteur Institute of Iran) were cultured in DMEM and RPMI-1640 media (Thermo Fisher Scientific, San Jose, CA, USA), respectively, containing penicillin and streptomycin and supplemented with 10% FBS (Gibco, Brazil, USA) at 37 °C a humidified atmosphere of 5% CO_2_ until 90% confluent. Cell line tests for mycoplasma, bacteria, and fungi contamination were negative.

### 4.2. Evaluation of Antitumor Activity

The antitumor efficacy of the peptides was investigated as described previously [11]. Thirteen days after implanting, tumor (~150 mm^3^)-bearing mice were grouped randomly into control and treated animals (6/group). From day 13 to day 33, the treatment group received cyclic or linear VGB3 (0.2 mg·kg^−1^), whereas the control group received an equal volume of PBS (i.v., once every two days). The volume of tumors was calculated as 0.52 × length × width^2^. In addition, changes in the weight of animals were recorded every 2 days.

### 4.3. Immunohistochemistry Analysis

For immunohistochemistry staining, deparaffinized tumor sections were incubated overnight at 4 °C with mouse monoclonal primary anti-Ki67 (5 µg·mL^−1^) or anti-CD31 (1:2000 (*v*/*v*)). The antigens were detected using 3,3-diaminobenzidine. All images were captured and analyzed with an Olympus BX51 microscope (Olympus America, New York, NY, USA).

### 4.4. VEGFR2 Binding Assay

A displacement assay was conducted as reported previously [51] with some modifications, including using 20 ng of the VEGFR2 extracellular domains (R&D Systems, Minneapolis, MN, USA). Peptide compounds were assayed three times per experiment.

### 4.5. Molecular Docking

PatchDock [52,53] and HADDOCK [54,55] were utilized for molecular docking study. The structure of ligands was generated using the MODELLER program, version 9.18, and refined using MD by the GROMACS, version 5.1.4; the VEGFR2 structure was extracted from the VEGFA/VEGFR2 complex structure (PDB code: 3V2A). To perform docking experiments, the PatchDock algorithm was used. The PatchDock performs molecular docking based on structure geometry and discovers docking transformations generating good molecular shape complementarity. Briefly, the PatchDock carries out three main steps, including (1) matching of surface patches, (2) representation of molecular shapes, and (3) filtering and scoring. Then, we further analyzed the top solutions by FireDock procedure and selected the best solutions. To evaluate the accuracy of the docked attitudes by PatchDock, we conducted a docking analysis employing the HADDOCK server. The solutions with the highest score and with the most negative atomic contact energy (ACE) value, which was also suggested by FireDock, were used for further assessment by the HADDOCK. The selected complexes were used for defining active and passive residues in the HADDOCK procedure. Next, two criteria, including the HADDOCK and Z score, were applied to discard redundant clusters and select the candidate cluster.

### 4.6. MTT Assay

The effects of C-VGB3 and L-VGB3 on the cell survival of HUVE and 4T1 cell lines were investigated using the MTT assay, as previously described [7], with some modifications. Cells were incubated with (0–1.81 μM) or without the peptide in the presence (20 ng·mL^−1^) or in the absence of VEGF (Sigma-Aldrich, St. Louis, MO, USA). After 24 and 48 h of incubation at 37 °C, the absorbance at 570 nm was recorded using an ELISA plate reader (Space Fax 2100, Awareness, Palm City, FL, USA). The tests were repeated three times.

### 4.7. Tubulogenesis Assay

Tube formation of HUVECs was assayed on the Matrigel [56]. After pre-labeling with PKH26 cell tracker (Sigma-Aldrich, St. Louis, MO, USA) HUVECs were plated into 24-well plates pre-coated with Matrigel Basement Membrane Matrix (BD Biosciences, San Jose, CA, USA). C-VGB3 (0.40 μM) was added to the medium, and images were manually captured after 12 h using an Olympus BX-51 fluorescence microscope. Quantitative analyses were performed by Wimasis online software.

### 4.8. Annexin V/PI Flow Cytometry

Annexin V/Propidium Iodide flow cytometry was performed to investigate the C-VGB3 effect on apoptosis induction, as previously described [33], with some modifications. The cultured HUVE and 4T1 cells were treated with PBS or C-VGB3 (0.40 μM) in the presence of 20 ng·mL^−1^ VEGF for 48 h, and then were analyzed using flow cytometry.

### 4.9. Western Blot Analysis

The levels of signaling proteins were investigated by the Western blot assay. 4T1 and HUVE cells stimulated by VEGF (20 ng·mL^−1^) were treated with (0.40 μM) or without C-VGB3 (treatment and control groups, respectively) after starvation in a serum-free medium for 24 h. After washing with PBS buffer twice, the cells were treated with RIPA lysis buffer containing (Sigma, St. Louis, MO, USA). Bradford′s test was performed to estimate the protein concentration. Equal amounts of total protein were electrophoresed on a 10% SDS-polyacrylamide gel, separated, and then transferred to a nitrocellulose membrane. Nitrocellulose was soaked in a blocking buffer containing 5% dry milk, Tris-buffered saline, and Tween 20 (TBST) for 1 h at ambient temperature. The nitrocellulose membrane was then washed with wash buffer and treated with antibodies against VEGFR2 p-VEGFR2, AKT, p-AKT, PI3K, mTOR, p-mTOR, NF-κB, p-NFκB, P53, MDM2, Bax, Bcl2, Bid, Bim, Apaf-1, cytochrome c, caspase-9, Fas (CD95), DR4 (CD261), DR5 (CD262), caspase3, caspase7, caspase8, PARP1, ERK 1/2, p-ERK1/2, Ras, Raf, Cdk4, GSK-3β, cyclin D1, vimentin, N-cadherin, E-cadherin, FAK, p-FAK, paxillin, p-paxillin, and β-actin overnight at 4 °C. The next steps were performed as described previously [25]. Because molecular weight ranges of target signaling proteins are very close, individual gels were run for separate signaling proteins (Figure S7).

### 4.10. Statistical Analysis

Data analysis production of graphs and statistical analysis were performed using the GraphPad Prism software (GraphPad Version8). Data were presented as mean ± SEM. For multiple comparisons, one-way ANOVA followed by Tukey’s post hoc test for statistical significance was used. A two-way ANOVA followed by Tukey’s post hoc test was used for in vivo efficacy experiments. *p*-values < 0.05 were considered statistically significant.

## 5. Conclusions

In conclusion, we showed that C-VGB3, which reproduces a binding region of VEGFB, could interfere with the interaction between VEGFA and VEGFR2 and inhibit angiogenesis and tumor growth via suppression of PI3K/AKT/mTOR and PLCγ/ERK1/2 pathways in both endothelial and tumor cells. In a broader context, our study suggests that the VEGFs share the binding regions that regulate their interaction with the ectodomain of their receptors. Identification of these common critical regions may be used to design molecules targeting more than one member of VEGFRs that are highly relevant in the pathogenesis of diverse cancers and blinding eye diseases.

## Figures and Tables

**Table 1:** Peptide-receptor docking results obtained by PatchDock and HADDOCK.

PatchDock					
	Complex	Score	Atomic Contact Energy (kcal·mol^−1^)		
	C-VGB3/VEGFR2	6572	−209.19		
	L-VGB3/VEGFR2	6280	−40.61		
**HADDOCK**					
Complex	Total interaction (kcal·mol^−1^)	Van der Waals (kcal·mol^−1^)	Electrostatic (kcal·mol^−1^)	Desolvation (kcal·mol^−1^)	Buried surface area (°A^2^)
C-VGB3/VEGFR2	−184 ± 26.9	−40.5 *±* 5.2	−143.5 *±* 21.7	0.9 *±* 6.1	961.7 *±* 33.0
L-VGB3/VEGFR2	−65.5 ± 15.8	−32.3 *±* 1.2	−33.2 *±* 14.6	−9.4 *±* 6.4	800.9 *±* 67.5

## Data Availability

The main data are included in this manuscript. All data are available from the corresponding author upon reasonable request.

## Supplementary Materials

The following supporting information can be downloaded at: https://www.mdpi.com/article/10.3390/ph16060906/s1. Figure S1. The Synthetic pathway for the synthesis of C-VGB3 sequence. The general method for the synthesis of C-VGB3 using SPPS. Figure S2. HPLC chromatogram of L-VGB3. Figure S3. HPLC chromatogram of C-VGB3. Figure S4. LC-MS of L-VGB3. Figure S5. LC-MS of C-VGB3. Figure S6. Evaluating the binding capability of L-VGB3 to VEGFR2. Figure S7. Raw data for western blot analysis. Table S1. Interaction of C-VGB3 and L-VGB3 with VEGFR2. The nature of interaction and participating residues are listed. Table S2. Peptide purification. Table S3. Analytical RP-HPLC separation [57,58,59,60,61,62].

## Abbreviations

bt-VEGF, biotinylated-VEGF; CDK, cyclin dependent kinase; DR, death receptor; DIEA, diisopropylethylamine; EC, endothelial cell; FFT, fast Fourier transforms; GSK3, glycogen synthase kinase 3; HUVEC, human umbilical vein endothelial cell; MCT, mammary carcinoma tumor; MVD, microvascular density; NRP1, neuropilin 1; PLCγ, phospholipase Cγ; PlGF, placenta growth factor; SD, steepest descent.
