# Supplementary material for: A VEGFB-Based Peptidomimetic Inhibits VEGFR2-Mediated PI3K/Akt/mTOR and PLCγ/ERK Signaling and Elicits Apoptotic, Antiangiogenic, and Antitumor Activities
Source: Pharmaceuticals (Basel). 2023 Jun 20;16(6):906. doi: 10.3390/ph16060906 (PMC10305181; doi:10.3390/ph16060906)
Supplement: Supplementary file 1 [file pharmaceuticals-16-00906-s001.zip › pharmaceuticals-2414566-supplementary.pdf]

Supplementary Material:

A VEGFB-Based Peptidomimetic Inhibits VEGFR2-Mediated PI3K/Akt/mTOR and PLCγ/ERK Signaling and Elicits Apoptotic, Antiangiogenic, and Antitumor Activities

Mohadeseh Namjoo[1], Hossein Ghafouri[2], Elham Assareh[2], Amir Reza Aref[3], Ebrahim Mostafavi[4], Ali Hamrahi Mohsen[5], Saeed Balalaie[6], Sylvain Broussy[7], S. Mohsen Asghari[5,*]

1- Department of Biology, University Campus II, University of Guilan, Rasht P.O. Box 14155-6619, Iran
2- Department of Biology, Faculty of Sciences, University of Guilan, Rasht P.O. Box 14155-6619, Iran; h.ghafoori@guilan.ac.ir (H.G.); assareh.elham7@gmail.com (E.A.)
3- Belfer Center for Applied Cancer Science, Dana-Farber Cancer Institute, Harvard Medical School, Boston, MA 02115, USA; amir@xspherabio.com
4- Department of Medicine, Stanford University School of Medicine, Stanford, CA 94305, USA; ebimsv@stanford.edu
5- Institute of Biochemistry and Biophysics (IBB), University of Tehran, Tehran P.O. Box 1841, Iran; hamrahi.ali.1992@gmail.com
6- Peptide Chemistry Research Center, K. N. Toosi University of Technology, Tehran P.O. Box 1841, Iran; balalaie@kntu.ac.ir
7- CiTCoM, UMR CNRS 8038, U1268 INSERM, UFR de Pharmacie, Faculté de Santé, Université Paris Cité,75006 Paris, France; sylvain.broussy@parisdescartes.fr
* Correspondence: sm.asghari@ut.ac.ir

This supporting information file contains:

- Supplementary Figure S1-S7
- Supplementary Table S1-S3
- Supplementary Methods
- Reference

Supplementary Figures:

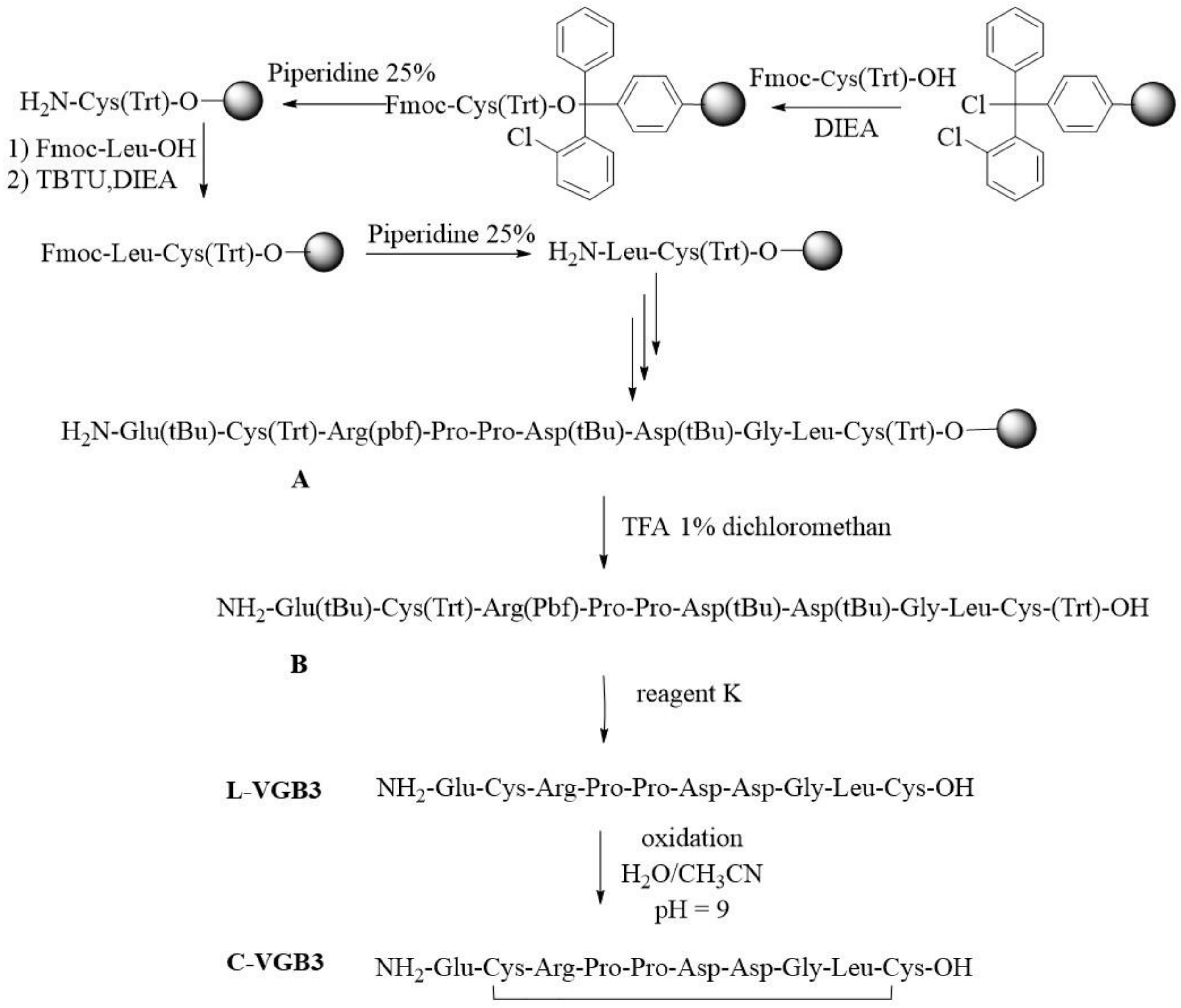

Figure S1. **The Synthetic pathway for the synthesis of C-VGB3 sequence.** The general method for the synthesis of C-VGB3 using SPPS.

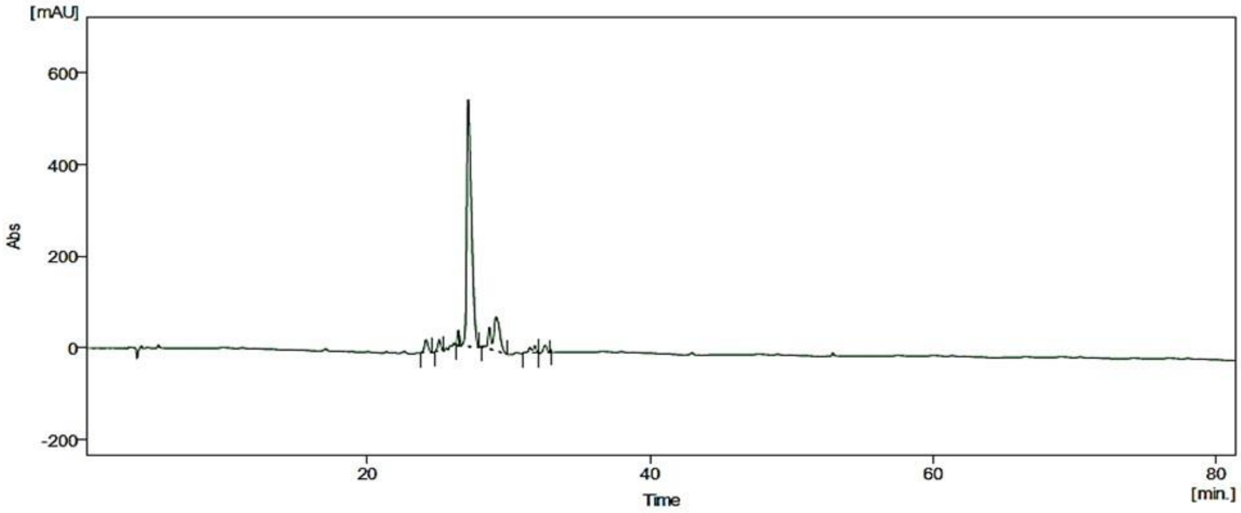

Figure S2. HPLC chromatogram of L-VGB3

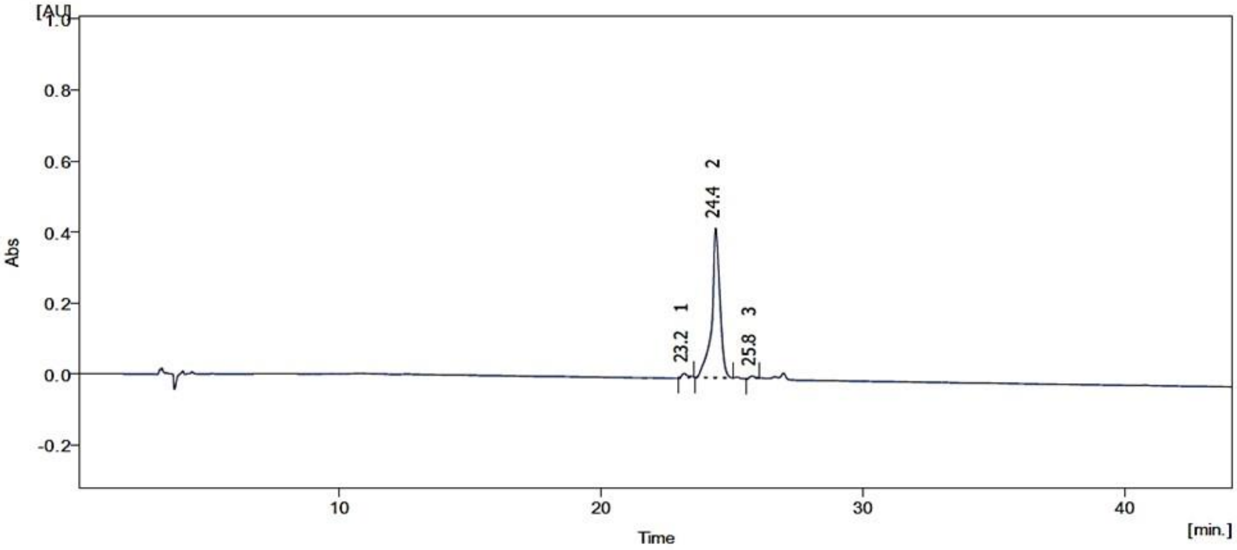

Figure S3. HPLC chromatogram of C-VGB3

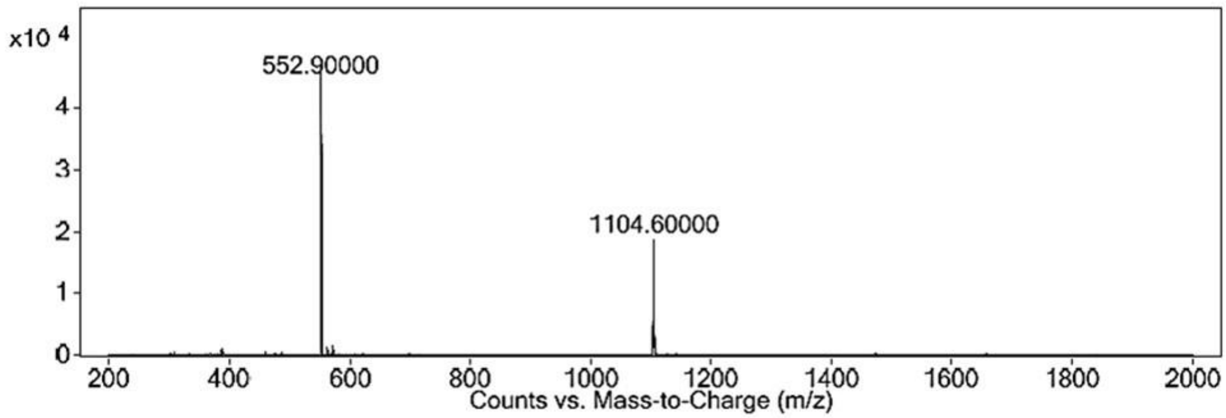

Figure S4. LC-MS of L-VGB3

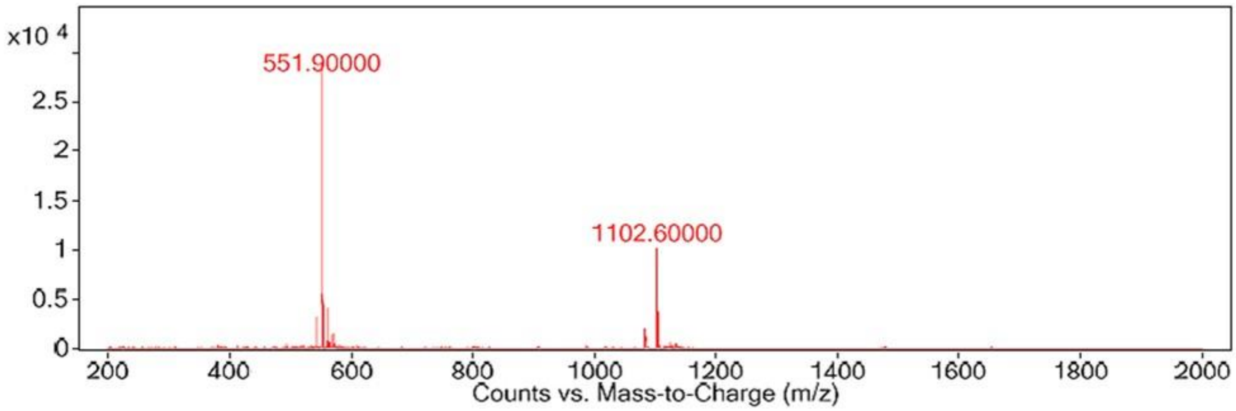

Figure S5. LC-MS of C-VGB3

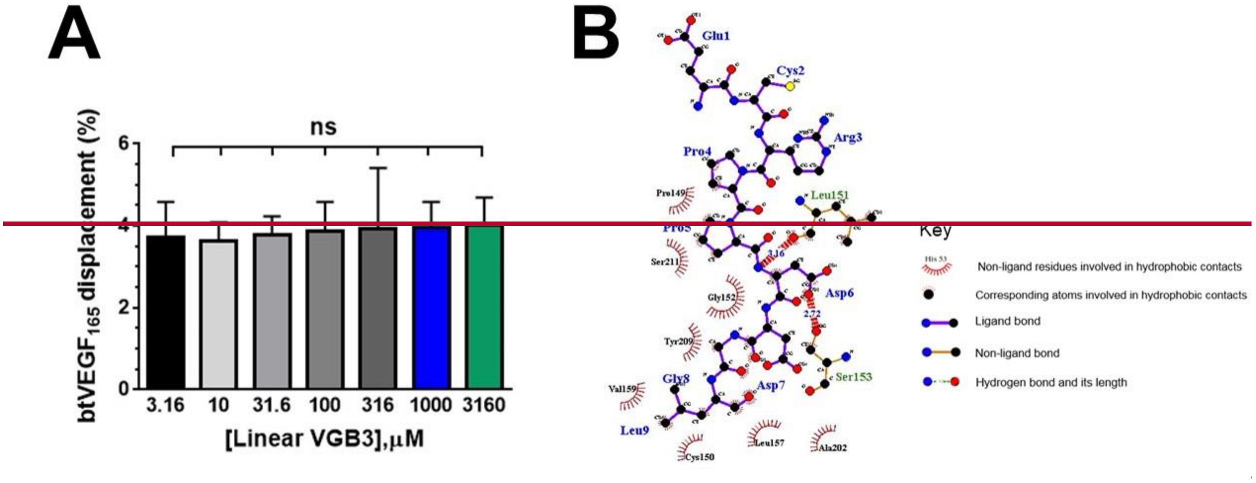

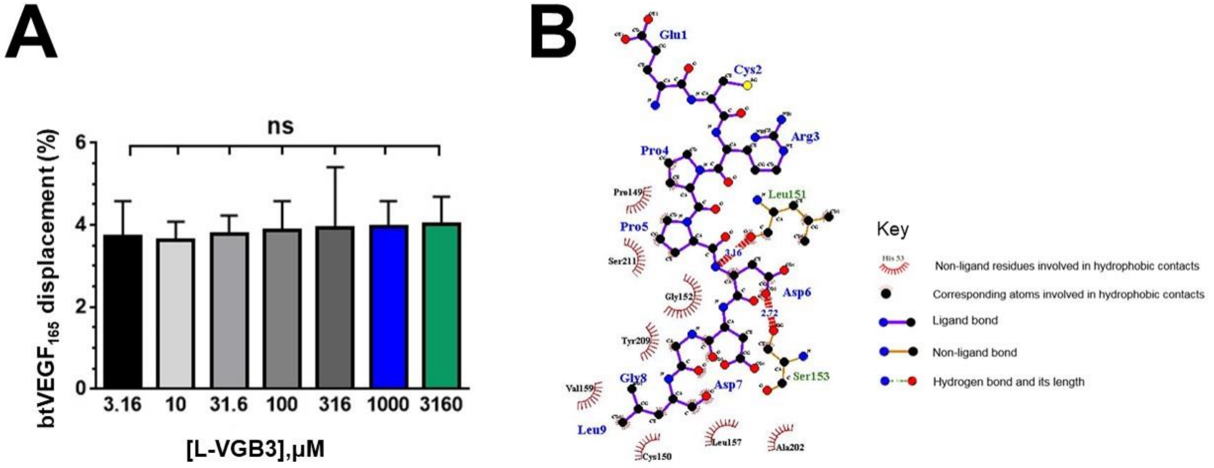

Figure S6. Evaluating the binding capability of L-VGB3 to VEGFR2

(A) ELISA-based displacement assay. The gradient concentrations of L-VGB3 (3.16, 10, 31.6, 100, 316, 1000 and 3160 μM) were used in the assay. L-VGB3 showed no binding to the extracellular domain of VEGFR2 at applied concentrations. **(B)** Two-dimensional representation of interactions (produced by LigPlot software) in L-VGB3/VEGFR2 complex.

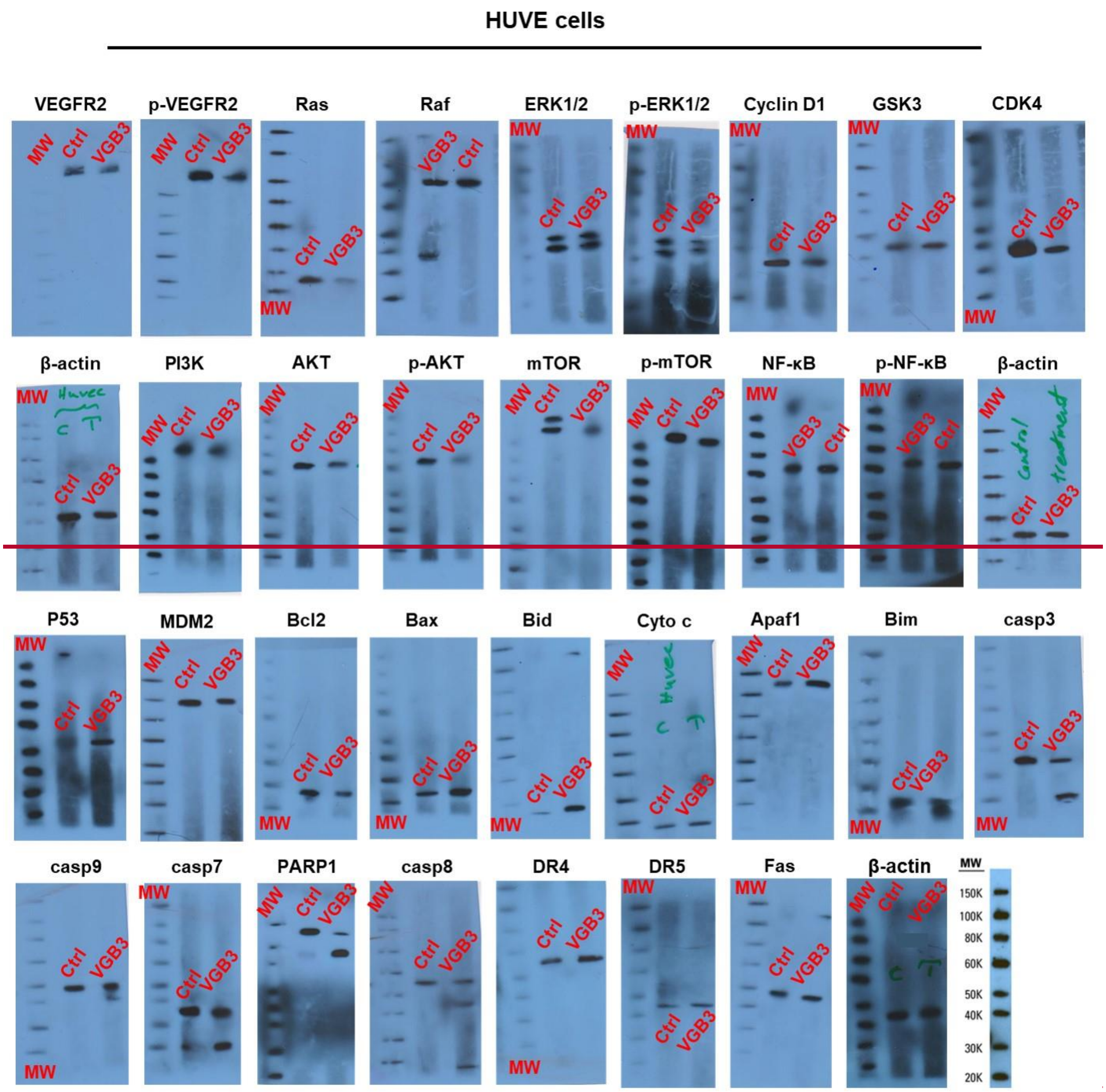
HUVE cells
VEGFR2
p-VEGFR2
Ras
Raf
ERK1/2
p-ERK1/2
Cyclin D1
GSK3
CDK4
β-actin
PI3K
AKT
p-AKT
mTOR
p-mTOR
NF-κB
p-NF-κB
β-actin
P53
MDM2
Bcl2
Bax
Bid
Cyto c
Apaf1
Bim
casp3
casp9
casp7
PARP1
casp8
DR4
DR5
Fas
β-actin
MW
Ctrl
VGB3
Huvec
C
T
control
treatment
150K
100K
80K
60K
50K
40K
30K
20K

HUVE cells

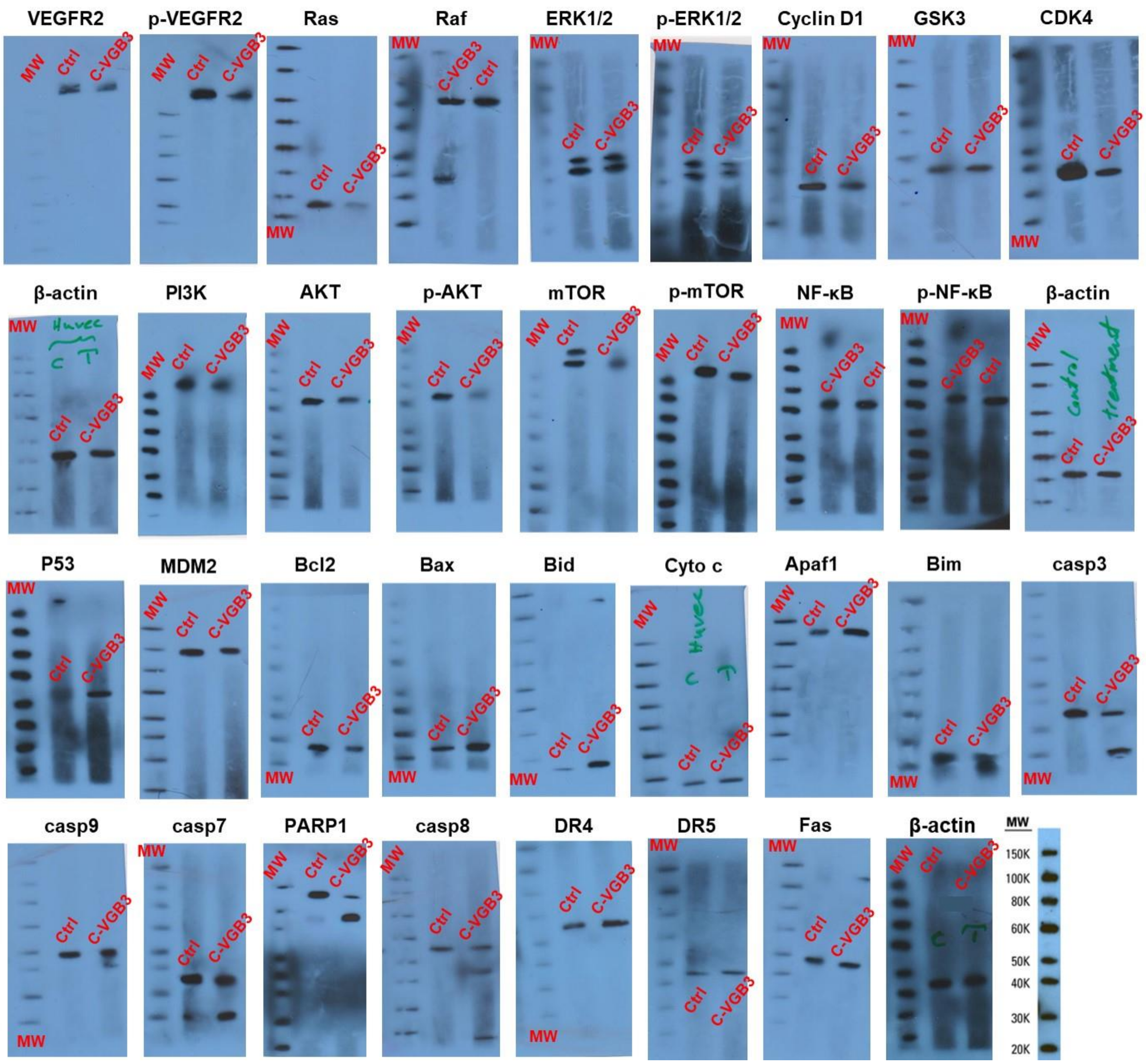

Figure S7

4T1 cells

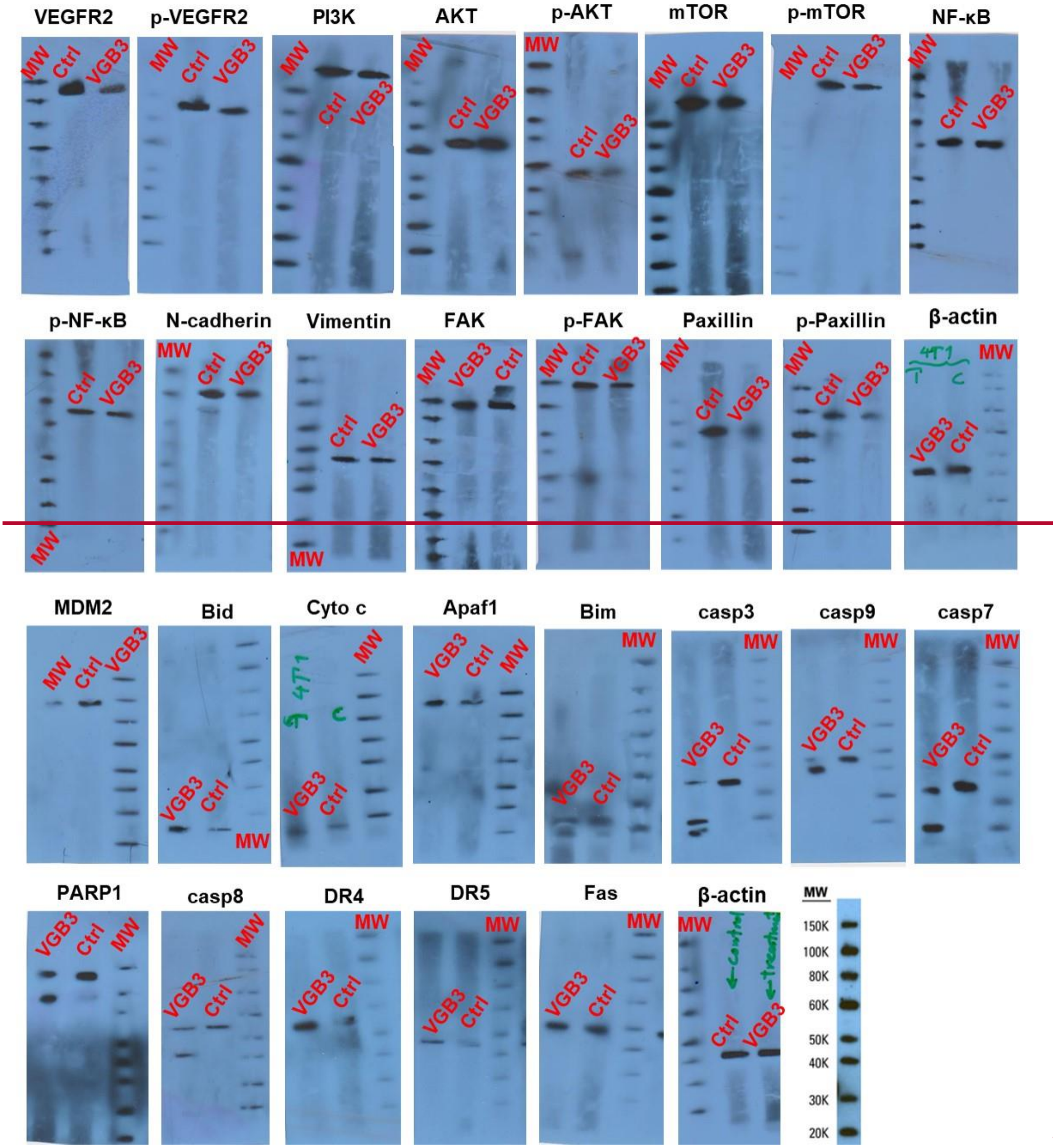
VEGFR2
p-VEGFR2
PI3K
AKT
p-AKT
mTOR
p-mTOR
NF-κB
p-NF-κB
N-cadherin
Vimentin
FAK
p-FAK
Paxillin
p-Paxillin
β-actin
MDM2
Bid
Cyto c
Apaf1
Bim
casp3
casp9
casp7
PARP1
casp8
DR4
DR5
Fas
β-actin
MW
Ctrl
VGB3
150K
100K
80K
60K
50K
40K
30K
20K

4T1 cells

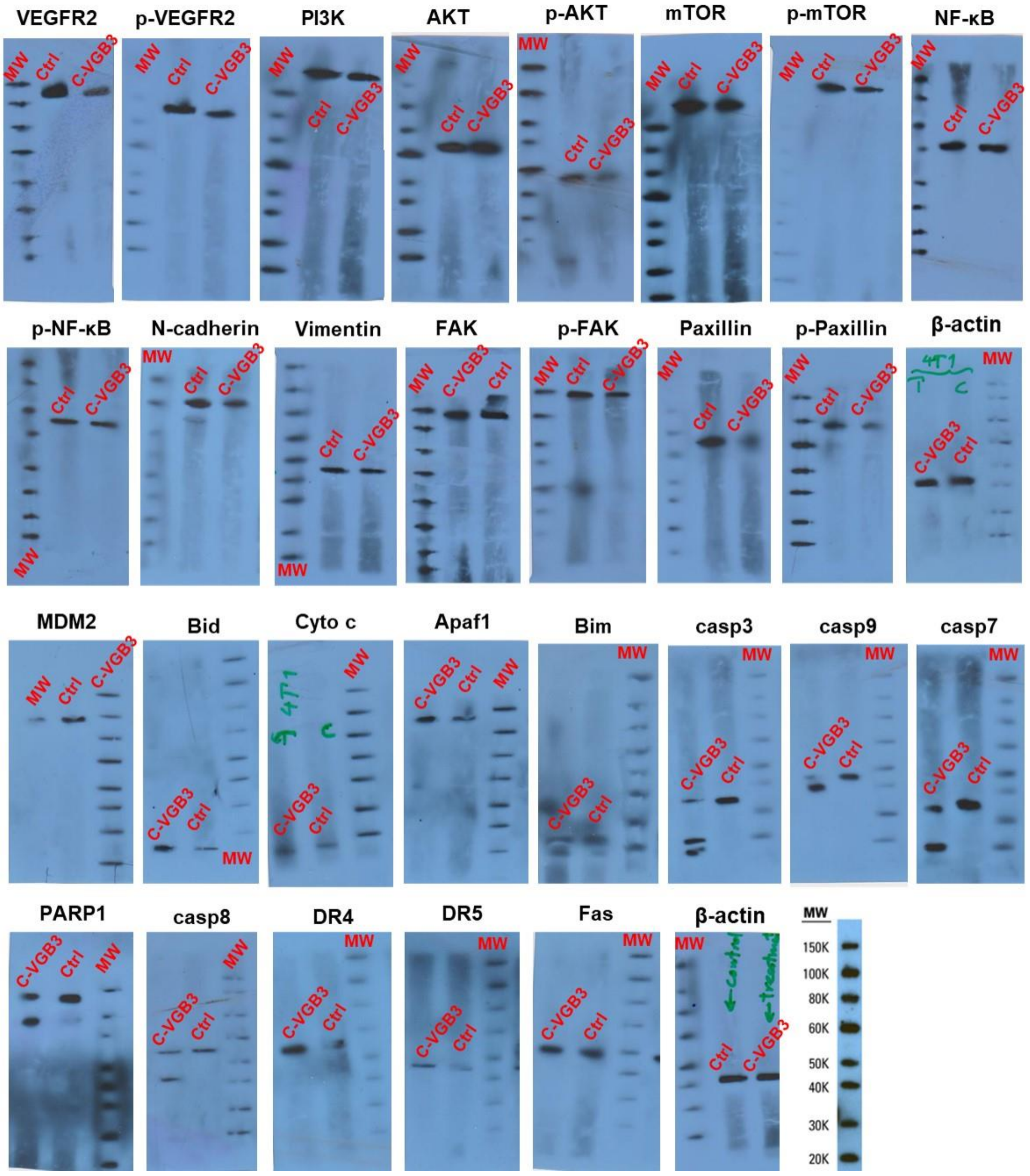

Figure S7. Raw data for western blot analysis.

Supplementary Tables:

Table S1. Interaction of C-VGB3 and L-VGB3 with VEGFR2. The nature of interaction and participating residues are listed.

Complex	Hydrogen bonds		Hydrophobic interactions
	Residue	Distance (Å)	
**C-VGB3/ VEGFR2**	C2 with Val135<br>D6 with Thr145<br>D6 with Lys144<br>D7 with Ser189<br>D7 with Thr145<br>D7 with Lys144<br><br>G8 with Tyr137<br>L9 with Tyr137<br><br>C10 with Val135	4.80<br>3.11<br>2.67<br>2.65<br>3.80<br>3.35<br>2.92<br>3.20<br>3.30<br>2.69<br>3.43	Ligand<br>C2, R3, P5, D6, D7, G8, L9, C10<br><br>Receptor<br>Val135, Val136, Tyr137, Ile138, Thr139, Lys142, Lys144, Thr145, Val146,Val147, Pro149
**L-VGB3/ VEGFR2**	D6 with Leu151<br>D6 with Ser153	3.16<br>2.72	Ligand<br>P4, P5, D6, D7, G8, L9<br><br>Receptor<br>Pro149, Cys150, Leu151, Gly152, Ser153, Val159, Tyr209, Ser211

Table S2. Peptide purification.

Entry	Flow (ml/min)	Time (min)	A%	B%
**1**	10	0	95	5
**2**	10	5	95	5
**3**	10	65	45	55
**4**	10	110	0	100

Table S3. Analytical RP-HPLC separation.

Entry	Flow (ml/min)	Time (min)	A%	B%
**1**	1	0	95	5
**2**	1	5	95	5
**3**	1	45	55	45
**4**	1	100	0	100

Supplementary Methods:

1. Reagents

Anti-AKT [(B-1): sc-5298], anti-p-AKT [(B-5): sc-271966], anti-PI3K [C2α (H-300): sc-67306], anti-mTOR [(30): sc-517464], anti-p-mTOR [(59.Ser 2448): sc-293133], anti-p-NF-κB [(A-8): sc-166748], anti-E-cadherin [(67A4): sc-21791], anti-P53 [(DO-1): sc-126], anti-MDM2 [(SMP14): sc-965], anti-Bax [(B-9):sc-7480], anti-Bcl2 [(N-19): sc-492], anti-Bim [(H-5): sc-374358], anti-caspase9 [(9CSP01): sc-81663], anti-cytochrome c [(A-8): sc-13156], anti-CD262 [(B-D37): sc-65314], anti-PARP1 [(F-2): sc-8007], anti-ERK 1/2 [(H-72): sc-292838], anti-p-ERK 1/2 [(Thr 177)-R: sc-16981-R], anti-Ras [(259): sc-35], anti-Raf-1 [(E-10): sc-7267], anti-Cdk4 [(DCS-35): sc-23896], anti-GSK-3β [(11B9): sc-81462], anti-Cyclin D1 [(A-12): sc-8396], anti-FAK [(D-1): sc-271126], anti-p-FAK [(2D11): sc-81493], anti-paxillin [(B-2): sc-365379], p-paxillin [(A-5): sc-365020], anti-β-actin [(C4): sc-47778], mouse anti-rabbit IgG-HRP (sc-2357) and m-IgGκ BP-HRP (sc-516102) were purchased from Santa Cruz Biotechnology INC, California, U.S.A. Anti-Vimentin (V9) and phycoerythrin (PE)-secondary anti-goat (P-2771MP) were purchased from Invitrogen, U.S.A. Anti-VEGFR2 (ab39256), anti-p-VEGFR2 (ab5473), anti-Bid (ab10640), anti-NF-κB (ab16502) and anti-CD261 (ab8414) were purchased from Abcam. Anti-Apaf-1 (E-AB-

12215) and anti-N-cadherin (E-AB-70061) were purchased from Elabscience ®. Anti-caspase7 (#9492), anti-caspase3 (#14220) and anti-caspase8 (#9746) were purchased from Cell Signaling Technology. Anti-CD95 (AF326) was purchased from R&D Systems. The molecular weight protein ladder (84785) was purchased from Thermo Scientific. Recombinant human VEGFR2/KDR Fc chimera protein (357-KD) was purchased from R&D Systems (Minneapolis, MN, U.S.A). TUNEL assays were done utilizing an *in situ* Cell Death Detection Kit POD (Roche Diagnostic GmbH, Germany). Amersham™ Protran® Premium nitrocellulose western blotting membranes (Ge10600013) was purchased from Sigma, U.S.A.

2. Peptide synthesis

Peptides were synthesized manually using standard Fmoc solid-phase peptide synthesis chemistry. The amino acids Fmoc-Cys(Trt)-OH, Fmoc-Leu-OH, Fmoc-Gly-OH, Fmoc-Asp(*t*Bu)-OH, Fmoc-Pro-OH, Fmoc-Arg(Pbf)-OH, Fmoc-Glu(*t*Bu)-OH were used. The peptide synthesis was carried out using 2-chlorotrityl chloride resin (1.0 mmol/g) following the standard Fmoc strategy. Firstly, the resin was swelled with DCM (3×10 mL) for 30 min. Then, Fmoc-Cys(Trt)-OH (0.586 g, 1.0 mmol) was attached to the 2-CTC resin (1.0 g) with DIPEA (1.36 ml, 8 mmol) in anhydrous DMF (10 mL) at room temperature for 3 h. After filtration, the remaining trityl chloride groups were capped by a solution of DCM/MeOH/DIPEA (20:2.4:1.2, 23.6 mL) for 30 min. The resin was filtered and washed thoroughly with DMF (3× 10 mL). After drying the resin under vacuum, the loading capacity was determined by weight and was 1.0. The resin-bound Fmoc-amino acid was washed with DMF (3× 10 mL) and treated with 25% piperidine in DMF (15 mL) for 30 min, followed by washing with DMF (3× 10 mL). Then, a solution of Fmoc-Leu-OH (0.708 g, 2.0 mmol), TBTU (0.64 g, 2.0 mmol), DIPEA (0.6 ml, 3.5 mmol) in 10 ml DMF was prepared, added to the resin-bound free amine, and shaken for 45 min at room temperature. After completion of the coupling, the resin was washed with DMF (3× 10 mL), followed by the coupling repeat for other amino acids of the sequence as the same method. The coupling was confirmed using Kaiser to detect the presence or absence of free primary amino groups in all cases. Fmoc concentration was determined by employing UV spectroscopy. The resin was washed

with DMF (3×10 mL) after completing all couplings, and the constructed linear peptide was cleaved from the resin by treatment with TFA (1%) in DCM (100 mL) and neutralization with pyridine (4%) in MeOH (50 mL). Then, the solvent was evaporated under reduced pressure and precipitated in water. 1 g of dry-protected decapeptide was treated with TFA/TES/H_2O/MeOH (95%: 2%: 1.5%: 1.5%) at room temperature for 2-3 h. The yield was 70% (0.773 g of linear VGB3).

The fully deprotected linear peptide was dissolved in H_2O or H_2O/acetonitrile. Then, the solution was buffered to pH=9 with ammonium acetate 10%, and the mixture was stirred at room temperature for 6 h. Afterward, the mixture was lyophilized and the cyclic peptide was analyzed by HPLC (high-performance liquid chromatography) and Mass (ESI).

The cyclic peptide was synthesized following the standard Fmoc-Solid Phase Peptide Synthesis (SPPS) procedure on 2-chlorotrityl resin and TBTU was used as a coupling reagent (Figure S1). Briefly, (1) Fmoc-Cys(Trt)-OH was loaded to the surface of the resin using diisopropylethylamine (DIEA); (2) after capping with MeOH other Fmoc-protected amino acids were added to the peptide sequence using TBTU as coupling reagent. Coupling reactions were performed utilizing Fmoc-amino acid/TBTU /DIEA/. A 25% piperidine in DMF solution was used to deprotect Fmoc groups. The Kaiser test was performed to confirm the coupling of amino acids. The desired fully protected decapeptide on the surface of resin A was obtained after the successive addition of the 10 amino acids in the sequence. The subsequent steps were performed to access linear decapeptide-based peptide: (1) the protected peptide A was cleaved from the resin surface utilizing 1% TFA, and compound B was formed; (2) the final deprotection was done using reagent K (TFA/TES/H2O/MeOH (95%: 2%: 1.5%: 1.5%); and (3) cyclization of the fully deprotected peptide was performed in 500 ml of H_2O or H_2O/acetonitrile (for 200 mg of linear peptide) with air oxidation to access the desired cyclic peptide. Subsequently, the solution was buffered to pH = 9 by adding ammonium acetate 10%. Cyclization was usually complete within 6 h, as shown by HPLC control. Then, the solvents were evaporated, and the peptides were lyophilized (Yields = 98 d). HPLC (Column C-18, Eurospher 100, 7 µm) was used to purify the peptide.

3. High performance liquid chromatography

The samples were dissolved in solvent A for liquid chromatography. The mobile phase for all HPLC purifications consisted of solvent A (Acetonitrile/Water (70/30)) and solvent B (NaH_2PO_4/Water (10 mM)), and separations were performed on an HPLC system (Knauer, Germany) equipped with a pump 1800 (Knauer, Germany), UV detector 2500 (Knauer, Germany) (Table S2). Peptide purification was performed at a preparative scale using the C18 column (120 mm × 20mm, 10 μm). The flow rate was set to 10 $mL.min^{-1}$ and the employed elution program started at 95 % A and remained at this point for 5 min before changing to 45 % of solvent A over 65 min. The elution profile was monitored via UV absorbance at 210 nm, and peptides were collected manually according to their absorbance at 210 nm.

Analytical RP-HPLC (Rigol, China) separation was carried out using an Agilent C18 column (250 mm × 4.6 mm, 5 μm) at a flow rate of 1 $mL.min^{-1}$ and the mobile phase consisted of solvent A [TFA/Water (0.1%)] and solvent B [Acetonitrile/Buffer A (80/20)]. Separations were performed on an HPLC system (Rigol, China) equipped with a pump 1000 (Rigol, China), UV detector 2500 (Rigol, China) (Table S3). The employed elution program started at 95 % A and remained at this point for 5 min before changing to 55 % of solvent A over 45 min at 1% min^{-1}. The elution profile was monitored via UV absorbance at 210 nm, and peptides were collected manually according to their absorbance at 210 nm. HPLC analysis found that linear decapeptide (H-Glu-Cys-Arg-Pro-Pro-Asp-Asp-Gly-Leu-Cys-OH) was obtained in 91 % < purity (t_R: 27.13 min) (Figure. S2). Also, HPLC analysis found that cyclic peptide was obtained in 96 % < purity (t_R: 24.38 min) (Figure S3).

4. Mass spectrometry analysis

The linear peptide structure was approved using Mass (ESI). Also, the cyclic peptide structure was approved using Mass (ESI). Mass (ESI): $C_{43}H_{69}N_{13}O_{17}S_2$ *m/z* = $[M+H]^+$ found 1104 for the linear peptide. Also, mass (ESI): $C_{43}H_{67}N_{13}O_{17}S_2$ *m/z* = $[M+ H]^+$ found 1102 for the cyclic peptide. LC-MS analyses were performed by an Agilent Triple Quadrupole LC/MS 6410 Diode array detector, ALS, TCC, Bin pump, and Degasser: 1200 series. The MS instrument was operated at the following settings:

Drying gas: Nitrogen (300 ^{o}C); source voltage 3.5 kV, nebulizing gas pressure: 50 PSI, Ionization mode: Electrospray. Product ions were then scanned and monitored (Figures S4 and S5).

5. Ellman assay

The Ellman assay was performed to confirm the presence of the disulfide bond in C-VGB3. The procedure includes the addition of the peptide (1.23 µM) into the sodium phosphate (100 mM) reaction buffer at pH 8.0 containing 100 mM EDTA; the addition of DTNB (4 $mg.mL^{-1}$) to the solution; a mix of the sample solution with an equal volume of the Ellman's reagent and incubation for 15 min at ambient temperature; and finally, read the absorbance of reaction at 412 nm (ε = 1.36 × 104 M^{-1} cm^{-1}) vs. a sulfhydryl standard 'cysteine' treated in the same manner to estimate the sulfhydryl concentration by a UV-3000 spectrophotometer (Pharmacia Biotech Ultrospect, Cambridge, England).

6. Molecular modeling and structural refinement

Models of C-VGB3 and L-VGB3 structures were built using homology modeling in the MODELLER software version 9.18 [1], and VEGFB (PDB ID: 2C7W) was used as a template. The VEGFB in FASTA format was obtained from the PDB website (http://www.rcsb.org/pdb/). Sequence alignments of each peptide with the template was directed using the multiple sequence alignment online server ClustalW (https://embnet.vital-it.ch/software/ClustalW.html). Ten models were created by MODELLER software, version 9.18, and the quality of the modeled structures was verified on the SWISS-MODEL web server (http://swissmodel.expasy.org/), PROCHECK [2] and QMEAN [3]. Molecular dynamics (MD) simulations were performed using the GROMACS package version 5.1.4 [4], to refine the best structures obtained from homology modeling. The 54A7 force field parameters [5] were used in the simulations and the interaction time steps during the simulation were set to 2 fs. The Particle-Mesh Ewald (PME) method with maximum Fast Fourier Transforms (FFT) grid spacing of 0.16 nm and an interpolation order of 4 was used to determine the long-range electrostatic interactions. A cutoff radius of 1.0 nm is used for Coulomb interactions and short-range interactions defined as van der Waals interactions [6].

Periodic boundary conditions were used, and bonds were restrained by applying the LINCS algorithm to their equilibrium position. Initial structures were solvated with three-point model water molecules in a cubic box of 1.0 nm to fully cover the protein system in water, following periodic boundary conditions (PBC). The produced system for each peptide was neutralized through the “genion tool” plugin of the GROMACS package by adding ions Na^+ and Cl^-. Then, the system was minimized for 50000 iterations using the steepest descent (SD) algorithm and using velocity rescaling (modified Berendsen) temperature coupling and Parrinello-Rahman pressure coupling methods, simulated under an isothermal-isobaric ensemble (NPT) for 5 ns at a constant temperature, the pressure of 300 K, and 1 atm, respectively. The NVT and NPT equilibration was carried out for 50000 steps. The minimization and equilibration procedures were executed, and finally, one system of the peptide was subjected to a 200 ns production run of MD simulations utilizing 2 fs as the time step and coordinates of all atoms were recorded in the form of .trr GROMACS trajectory file every 10 ps.

7. TUNEL assay

2×10^3 HUVE or 4T1 cells were cultured in DMEM and RPMI-1640 media, respectively, supplemented with 10% (v/v) FBS overnight. Then, the cells were incubated in the media supplemented with 5% FBS, with PBS or C-VGB3 (0.40 μM). The terminal deoxynucleotidyl transferase (TdT) dUTP nick-end labeling (TUNEL) assay was performed on the HUVE and 4T1 cells using TUNEL Assay Kit (FITC or HRP-DAB; E-CK-A332) according to manufacturer instructions. The steps included: (1) washing the cells once with PBS; (2) fixation of the HUVE or 4T1 cells in fixative buffer at room temperature for 15-60 min; (3) washing the HUVE or 4T1 cells with PBS for 5 min (three times); (4) adding blocking buffer on the slides, and incubation at room temperature for 10 min; (5) repeating the step 3; (6) putting the slides into the permeabilization buffer on an ice bath for 2 min, and (7) repeating the step 3. The following steps were performed for TUNEL staining: (1 washing the slides with PBS (2 times, 5 min each time) and wipe drying the liquid on the slides; (2) adding 50 μL TdT enzyme working solution to each slide, and incubating at 37°C (60 min in a wet bow); (3) washing the slide with PBS (3 times, 5 min each time); (4) wipe drying the

liquid on the slides with absorbent paper; (5) adding DAPI, and incubating at room temperature for 5 min; (6) washing the slides with PBS (4 times, 5 min each time) and wipe drying the liquid on the slides with absorbent paper; and (7) adding mounting medium (contain Fluorescent Mounting Media) to seal the slides. Fluorescence microscopy was done for detection according to manufacturer instructions. Image analysis was done using ImageJ software.

References

1. Eswar, N., Webb, B., Marti-Renom, M. A., Madhusudhan, M., Eramian, D., Shen, M. y., Pieper, U. & Sali, A. (2006). Comparative protein structure modeling using Modeller. *Curr. Protoc. Bioinformatics* **15**, 5.6. 1-5.6. 30.

2. Laskowski, R. A., MacArthur, M. W., Moss, D. S. & Thornton, J. M. (1993). PROCHECK: a program to check the stereochemical quality of protein structures. *J. Appl. Crystallogr.* **26**, 283-291.

3. Benkert, P., Tosatto, S. C. & Schomburg, D. (2008). QMEAN: A comprehensive scoring function for model quality assessment. *Proteins: Structure, Function, Bioinformatics*. **71**, 261-277.

4. Abraham, M. J., Murtola, T., Schulz, R., Páll, S., Smith, J. C., Hess, B. & Lindahl, E. (2015). GROMACS: High performance molecular simulations through multi-level parallelism from laptops to supercomputers. *SoftwareX*. **1**, 19-25.

5. Schmid, N., Eichenberger, A. P., Choutko, A., Riniker, S., Winger, M., Mark, A. E. & van Gunsteren, W. F. (2011). Definition and testing of the GROMOS force-field versions 54A7 and 54B7. *Eur. Biophys. J.* **40**, 843.

6. Tao, Y., Rao, Z.-H. & Liu, S.-Q. (2010). Insight derived from molecular dynamics simulation into substrate-induced changes in protein motions of proteinase K. *J BIOMOL STRUCT DYN* **28**, 143-157.
